# Multilayered control of exon acquisition permits the emergence of novel forms of regulatory control

**DOI:** 10.1186/s13059-019-1757-5

**Published:** 2019-07-17

**Authors:** Nesli Avgan, Juli I. Wang, Javier Fernandez-Chamorro, Robert J. Weatheritt

**Affiliations:** 1 EMBL Australia, Garvan Institute of Medical Research, Sydney, NSW 2010 Australia; 2 St. Vincent’s Clinical School, University of New South Wales, Sydney, NSW 2010 Australia

**Keywords:** Alternative splicing, Gene regulation, Evolution, DNA damage, Exonization, Cancer

## Abstract

**Background:**

The long introns of mammals are pools of evolutionary potential due to the multiplicity of sequences that permit the acquisition of novel exons. However, the permissibility of genes to this type of acquisition and its influence on the evolution of cell regulation is poorly understood.

**Results:**

Here, we observe that human genes are highly permissive to the inclusion of novel exonic regions permitting the emergence of novel regulatory features. Our analysis reveals the potential for novel exon acquisition to occur in over 30% of evaluated human genes. Regulatory processes including the rate of splicing efficiency and RNA polymerase II (RNAPII) elongation control this process by modulating the “window of opportunity” for spliceosomal recognition. DNA damage alters this window promoting the inclusion of repeat-derived novel exons that reduce the ribosomal engagement of cell cycle genes. Finally, we demonstrate that the inclusion of novel exons is suppressed in hematological cancer samples and can be reversed by drugs modulating the rate of RNAPII elongation.

**Conclusion:**

Our work demonstrates that the inclusion of repeat-associated novel intronic regions is a tightly controlled process capable of expanding the regulatory capacity of cells.

**Electronic supplementary material:**

The online version of this article (10.1186/s13059-019-1757-5) contains supplementary material, which is available to authorized users.

## Background

A major challenge in biology is to understand how complex regulatory networks emerge during evolution. An important mechanism for expanding complexity is alternative pre-mRNA splicing (AS), the process by which exonic regions are differentially excised to create multiple transcripts from a single gene locus. Recent surveys of organ transcriptomes across several vertebrate species have revealed AS profiles have diverged rapidly during vertebrate evolution, whereas organ mRNA expression profiles have remained relatively conserved [1, 2]. Moreover, several studies have described examples of how the emergence of lineage-specific isoforms can create novel phenotypes [3–5].

However, the emergence of AS-dependent complexity comes at a cost. On the one hand, AS confers flexibility to gene function by altering reading frame and tuning transcript stability [1, 4, 6–8]. On the other hand, the inappropriate recognition of intronic sequences resembling splice sites can give rise to the non-canonical execution of regulatory events disrupting gene expression [9]. These deleterious events often manifest themselves within human diseases [10]. Despite the importance of AS in evolution, the mechanisms and genomic features that control this balance between the promotion of novel functionality and its prevalence to cause disease are poorly understood.

In this study, we surveyed the human transcriptome to identify thousands of novel exonization events, the process by which non-canonical intronic sequences are incorporated into mRNA transcripts. We reveal these events do not occur randomly within the genome but are enriched within cell cycle and cell signaling genes. Exonization events occur within m6a (N^6^-methyladenosine)-modified long introns close to the transcription start site and often overlap Alu and L1 transposon events. The inclusion of these novel exons is promoted by regulatory events that promote the “window of opportunity” for spliceosome recognition, such as the rate of RNA polymerase II elongation and splicing efficiency dynamics. This multilayered system can be actively regulated by exogenous agents permitting the emergence of novel regulation as exemplified by UV irradiation, which promotes exonization within cell cycle genes to suppress their ribosomal engagement. We further provide evidence exonization is suppressed in hematological cancers. Thus, we identify a highly evolvable mechanism that can expand the regulatory complexity of cells.

## Results

### Exonization events occur in introns enriched with new transposons

**Fig. 1:**
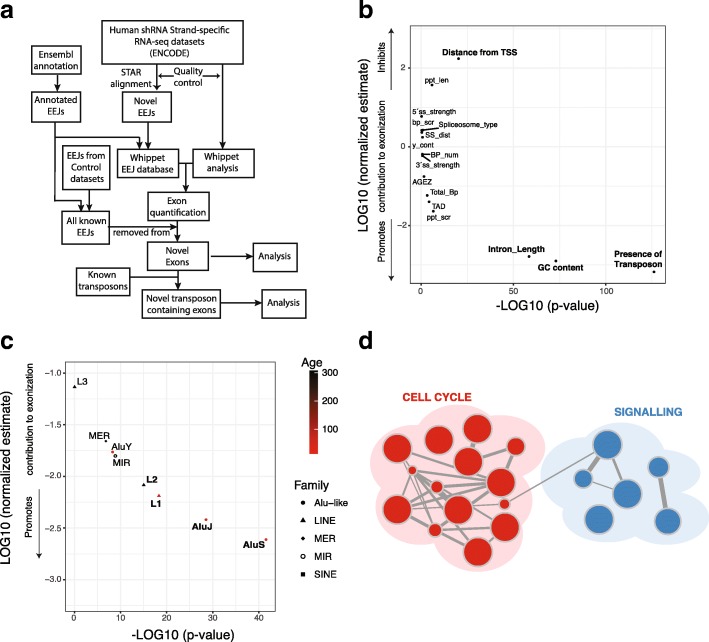
Genomics features of introns with exonization events. **a** Workflow to identify novel exonization events (see the “Methods” section). Briefly, RNA-seq from shRNA knockdown of RNA binding proteins in HepG2 is analyzed by 2-pass enabled STAR, and then novel junctions are incorporated into index files analyzed by Whippet [12]. Identified exons are filtered to remove exon-exon junctions and events occurring in any of the matched control samples, as well as annotated in genome databases. Only events supported by > 5 reads mapping over exon-exon junctions and a percent spliced in (PSI) greater than 5% are included. **b** Plot showing the results from a logistic linear regression analysis aimed at identifying features important in discriminating introns prone to exonization events to all other expressed introns. Features in bold significantly contribute to the model (*p* < 0.01, Student *t* test). TSS, transcription start site; ppt_len, polypyrimidine tract length; 5′ss, 5′-splice site; 3’ss, 3′-splice site; bp_scr, branchpoint score; SS_dist, splice site distance; BP_num, branchpoint number; AGEZ, AG dinucleotide Exclusion Zone length; TAD, topologically associating domain; ppt_scr, polypyrimidine tract score (*n* = 13,103). **c** Plot showing the results from a logistic linear regression analysis aimed at identifying the type of transposable elements that most effectively discriminate introns prone to exonization events compared to all other expressed introns. Features in bold significantly contribute to the model (*p* < 0.01, Student *t* test). Nodes are colored by average estimated age of when transposable elements arose (*n* = 13,103). **d** Enrichment map for GO, REACTOME, and KEGG functional categories of genes that contain Alu-exonization events, with representative GO terms shown for each sub-network (see Additional file 1: Figure S1 for annotated version). Node size is proportional to the number of genes associated with the GO category, and edge width is proportional to the number of genes shared between GO categories

To investigate the potential for novel exonization events to occur within the human transcriptome, we analyzed over 400 shRNA knockdown RNA-seq datasets from HepG2 cell lines [11] to identify reads mapping between known exons and novel intronic sequences (Fig. 1a and the “Methods” section). We only considered reads mapping to exon-exon junctions (EEJs) supported by at least 5 reads and a percent spliced in (PSI) value of at least 5%. Novel exons were defined as those absent from annotation databases [13, 14] and all non-perturbed control datasets (Fig. 1a). Confirming the validity of this approach, the knockdown of the RNA binding protein (RBP) heterogeneous ribonucleoprotein C (hnRNPC) created the most Alu-derived exonization events (Additional file 1: Figure S1), in line with previous observations [15]. In total, we detected 13,103 novel exonic events within 4774 genes or 30.6% of evaluated human protein-coding genes under the perturbations we surveyed.

To investigate the mechanisms underlying these exonization events, we collated a list of features classically associated with alternative splicing, including splice site strength, GC content, and polypyrimidine tract length (Additional file 2: Table S1). Logistic linear regression was then used to compare these events with a “background” group of expressed introns lacking any evidence of exonization (Fig. 1b). Validating our choice of genomic features, our model achieves a high average true positive rate [AUC, area under the receiver operating characteristic (ROC) curve] of 75.2% (Additional file 1: Figure S1). Moreover, we were able to confirm previous results that exonization events tend to occur in long introns with a high GC content [16] (Additional file 1: Figure S1, intron length: *p* < 3.53 × 10^−59^, GC 1.01 × 10^−73^, Student *t* test). Notably, we find exonization events often overlap nucleosome-binding sites (Additional file 1: Figure S1, *p* < 2.37 × 10^−23^, Wilcoxon test), rarely occur at the 3´-end of the gene body (*p* < 5.90 × 10^−21^, Student *t* test) and show a significant tendency to occur within 5´-UTRs (Additional file 1: Figure S1, *p* < 7.13 × 10^−166^, Fisher exact test). Importantly, we also observe that the strongest predictor for exonization was the occurrence of transposable elements overlapping the novel exon (Fig. 1b and Additional file 1: Figure S1, *p* < 6.46 × 10^−127^, Student *t* test). In comparison to these strong predictors, no cis-regulatory splicing elements contribute significantly to the model or show significant differences between the datasets (Additional file 1: Figure S1, *p* > 0.05, Student *t* test).

To evaluate the conservation of novel exon usage across species, we analyzed the extent of exonization across multiple matched tissue types within four primate species spanning 30 million years of primate evolution. To explore exonization usage between samples, genes with events occurring in all four species were identified and sorted using affinity propagation clustering. In line with canonical alternative splicing [1, 2], samples from the same species invariably clustered together (Additional file 1: Figure S2). The notable exception to this trend was observed in samples from the testes, which showed tissue-specific clustering. This suggests within the testes there is a strong exonization signature conserved across primate species in multiple genes (Additional file 1: Figure S2).

To further investigate the influence of retrotransposons on exonization, we sub-divided the transposable elements into their major sub-families. In line with the lineage-specificity of exonization events, the most significant contributors to the model are transposons younger than 70 million years. In particular, Alu element sub-family members AluJ and AluS, as well as the highly mobile L1 elements are strong predictors (Fig. 1c). Interestingly, an exception to this rule is the AluY sub-family [17], which shows a pattern reminiscent of much older transposon events (Fig. 1c). This difference is potentially due to its relative depletion within gene bodies (3%, 19%, AluY, AluS, occurrence in expressed introns). Finally, we wished to assess which type of genes contains Alu-exonization events. Interestingly, we find a strong enrichment for functions related to cell signaling and cell cycle regulation (Fig. 1d, Additional file 1: Figure S1 and Additional file 3: Table S2). Alongside previous examples [8, 9, 15, 18], our observation of an extensive number of exonization events overlapping new transposons suggests a novel source of transcriptomic complexity.

### m6a RNA binding proteins suppress exonization

**Fig. 2:**
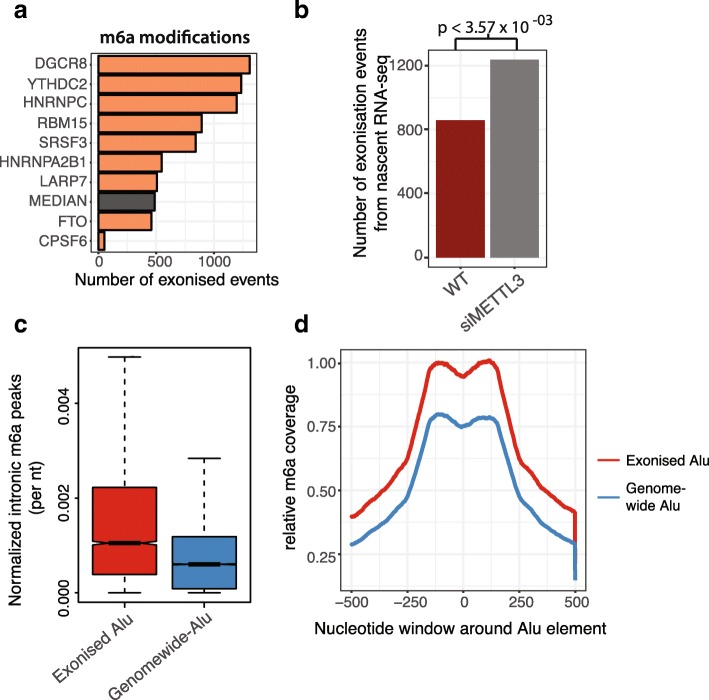
m6a methylation suppresses exonization. **a** Barplots of m6a-methylation associated genes and the number of novel exons identified upon knockdown in HepG2 cells **b** Barplot showing number of exonization events induced upon knockdown of N6-adenosine-methyltransferase (METTL3) compared to a control sample. *p* value calculated using Wilcoxon-rank sum test. **c** Boxplots displaying normalized intronic m6a peaks per nucleotide from nascent RNA in HepG2 cells. Genomewide Alu elements occur within expressed introns with no identified exonization events. Boxplots display the interquartile range as a solid box, 1.5 times the interquartile range as vertical thin lines, the median as a horizontal line, and the confidence interval around the median as a notch. nt, nucleotide (*n* = 13,247). **d** Plot showing relative m6a coverage in nucleotides surrounding Alu elements. See **c** for the description of “genome-wide”. (*n* = 13,247)

An evaluation of the trans-factors promoting exonization (Additional file 1: Figure S2 and Additional file 4: Table S3) revealed an enrichment of m6a (N^6^-methyladenosine) binding RBPs, especially among Alu-containing novel exons (Fig. 2a, *p* < 0.05, hypermetric test). This included hnRNPC, which has been previously shown to induce a large number of Alu-specific exonization events [15], as well as DiGeorge syndrome critical region 8 (DGCR8) and YTH domain-containing protein 2 (YTHDC2). To examine the potential role of m6a marks in exonization, we analyzed knockdown data of the m6a modification enzyme N6-adenosine-methyltransferase subunit (METTL3) [18]. This analysis revealed a significant increase in the number of detectable exonization events upon METTL3 knockdown (Fig. 2b, *p* < 3.57 × 10^−03^, Wilcox-rank sum test), in concordance m6a regulating the inclusion of novel exons. Further analysis of these METTL3-dependent exonization events revealed a functional enrichment of genes associated with DNA damage (*p* < 2.68 × 10^−02^, FDR-corrected *p* value). Next, we analyzed data from HeLa cells constituting of two knockdowns of known m6a regulators (Serine/arginine-rich splicing factor 3 **(**SRSF3) and YTH domain-containing protein 1 **(**YTHDC1)) and two knockdowns of RBPs not known to directly recognize m6a (Serine/arginine-rich splicing factor 9 and 10 (SRSF9 and SRSF10)) [19, 20]. In agreement with previous results, we find that the knockdown of either SRSF3 or YTHDC2 strongly induces exonization whereas decreasing the expression of SRSF9 or SRSF10 has little or no impact (Additional file 1: Figure S2).

To further assess whether m6a modification impacts exonization events, we assessed the enrichment of m6a sites within exonized sequences [21]. This approach used BrU-seq followed by isolation of m6a-methylated fragments using an m6a-specific antibody [21]. Due to the repetitive nature of Alu-elements, we used expectation maximization to assign multimapping reads (maximum of 10 matches allowed) to Alu-elements based on the expression of the host gene [22]. As a comparison set, we examined all Alu-elements within expressed intronic regions that are not in the vicinity of a novel exon (see the “Methods” section). This analysis revealed a strong enrichment of m6a sites mapping to exonized Alu-elements (Fig. 2c, *p* < 3.23 × 10^−123^, Wilcoxon rank-sum test; Fig. 2d, *p* < 2.13 × 10^−63^, Wilcoxon rank sum test; Additional file 1: Figure S2). Altogether, this suggests m6a modification and binding proteins are key regulators of (Alu-containing) exonization events.

### Mechanisms increasing the “window of opportunity” for spliceosome recruitment promote exonization

**Fig. 3:**
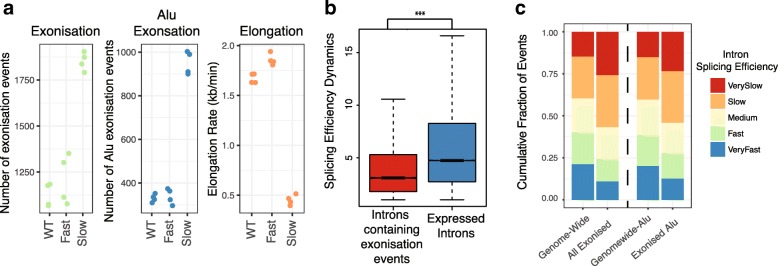
Reduced rate of RNA polymerase II elongation and poor splicing efficiency promotes exonization. **a** Dot plot showing the impact of RNA polymerase mutations on exonization of Alu-containing exons. WT, wildtype; Fast, E1126G mutation; Slow, R749H mutation. Each point represents individual dataset. KB, kilobase; min, minutes. Elongation, rate of transcriptional elongation (see the “Methods” section and [24]). **b** Boxplot of splicing efficiency for introns with exonization events vs all expressed introns with no evidence of exonization. Splicing efficiency is a metric describing speed of intron excision as measured by assessing nascent RNA-seq using BrU-chase at 0, 15, 30, and 60 min. See Fig. 2d for the description of boxplots. ***p < 1 × 10^−10^ *p* value calculated using Wilcoxon-rank sum test. (*n* = 4,011). **c** Stacked bar plot showing distributions of introns for splicing efficiency identified by BrU-chase. Groups assigned by *K*-means clustering (*k* = 5) (see the “Methods” section)—see Additional file 1: Figure S3a for distributions of splicing efficiencies. (*n* = 83,972)

Our initial analysis revealed the prediction of exonization is strongly enhanced by repeat elements, intron length and GC content. These features are known to negatively correlate with RNA polymerase II (RNAPII) elongation rate [23]. We therefore hypothesized that changes in RNAPII elongation rate may promote exonization. To test this, we analyzed RNA-seq data from human cells expressing mutations that increase (E1126G) or decrease (R749H) the elongation rate of RNA polymerase II (RNAPII) [24]. To determine the elongation rate for each mutant, we analyzed data from genome-wide nuclear run-on sequencing (GRO-seq) assay combined with transcription elongation inhibitor DRB (see the “Methods” section and [24]). We observe that mutations slowing the rate of RNAPII elongation (R749H) strongly induced exonization events (Fig. 3a, ALL: *p* < 3.18 × 10^−45^, Fisher’s exact test), with this change especially strong in Alu-containing novel exons (Fig. 3a, ALU: *p* < 5.62 × 10^−53^, Fisher’s exact test). In contrast, mutations that speed up elongation had negligible effects on the number of exonization events detected (Fig. 3a; *p* > 0.05, Fisher’s exact test).

This result supports the competition model of alternative splicing [24, 25] wherein the regulation of exon inclusion is associated a “window of opportunity” for spliceosome recognition. If this connection between exonization and opportunity is valid, an independent mechanism for the emergence of novel exons should occur within introns that are slowly processed by the splicing machinery. To evaluate this hypothesis, we analyzed nascent RNA data from BrU-Chase-seq [21], in which cells are labeled with a 15-min BrU pulse and chased for 0, 15, 30, and 60 min. To determine splicing kinetics for each intron, we calculated the splicing efficiency dynamics (SEDs) or the rate of intron excision [21, 26] (see the “Methods” section). *K*-means clustering was then used to identify five groups of introns with SEDs ranging from very fast to very slow (Additional file 1: Figure S3). Introns containing exonization events were then compared to a background set of all expressed introns. Strikingly, this analysis reveals that introns containing exonization events are strongly enriched within the slowest SED cluster (Fig. 3b, *p* < 3.23 × 10^−239^, Wilcox rank-sum test, and Additional file 1: Figure S3). Moreover, these introns display a highly significant reduction in SED compared to background groups (Fig. 3c and Additional file 1: Figure S3, *p* < 2.2 × 10^−160^, Wilcoxon rank-sum test). Together, these observations suggest that mechanisms that expand the “window of opportunity” will increase the likelihood of recognition by the splicing machinery and thereby promote the rate of exonization.

### DNA damage induces exonization within cell cycle genes

**Fig. 4:**
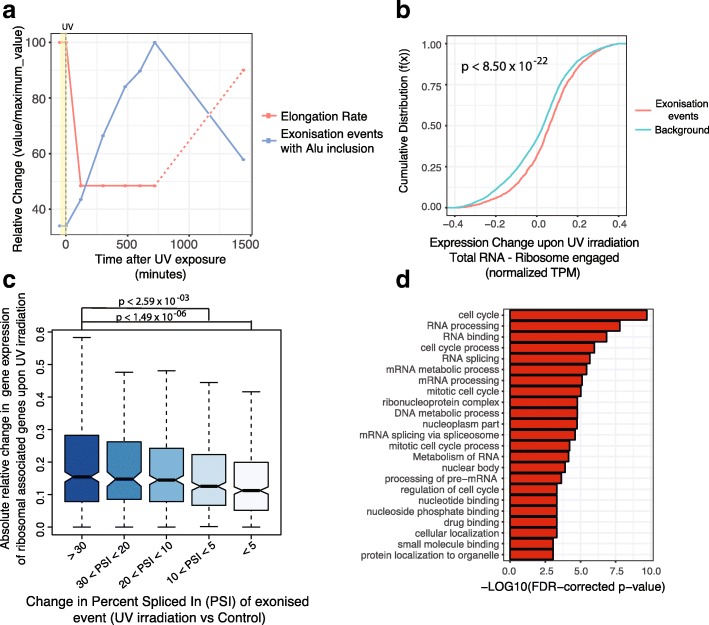
UV irradiation increases the number of exonization within cell cycle genes promoting transcript retention in nucleus. **a** Line plot displaying the results of GRO-seq analysis following UV (ultraviolet) irradiation. The dotted line represents estimate based on data from the original paper [28]. See the original data in Additional file 1: Figure S4. The yellow shaded region represents UV application (*n* = 3,278). **b** Cumulative distribution plot of change in expression of genes within polysome fraction compared to whole cell fraction. A cumulative distribution plot describes the proportion of data (*y*-axis) less than or equal to a specified value (*x*-axis). Cumulative Distribution *F*(*x*), cumulative distribution function. *p* value calculated using Wilcoxon-rank sum test. **c** Boxplots showing normalized changes (change in TPM/max (TPM)) in the difference of expression between total RNA-seq and ribosomal-engaged RNA-seq after UV irradiation. Genes are binned by percent spliced in (PSI) increase of exonized novel exon after UV irradiation. Bin sample size from left to right: *n* = 427, 158, 355, 410, and 438. See Fig. 2d for the description of boxplots. *P* values calculated using Wilcoxon-rank sum test. TPM, transcripts per million. **d** Functional categories of genes that undergo exonization upon UV irradiation compared to control dataset (also see Additional file 1: Figure S4). FDR, false discovery rate

Exogenous process can also promote alterations in transcription elongation and therefore may alter rates of exonization. To investigate this we focused on UV irradiation as previous studies have demonstrated that it promotes both the hyperphosphorylation of RNAPII leading to the subsequent inhibition of transcription elongation [27, 28] and the recruitment of the m6a machinery to sites of DNA damage [29]. Using data obtained from nascent RNA-seq (GRO-seq protocol), we evaluated the impact of UV irradiation on Alu-exonization over a 24-h period [28]. Remarkably, following the steep decrease in the rate of RNAPII elongation upon UV irradiation we find an equally striking sharp increase in the rate of exonization (Fig. 4a and Additional file 1: Figure S4). This incorporation of novel exons continues to rise as long as the RNAPII elongation rate remains low. Importantly, the full recovery of the RNAPII elongation rate at the 24-h mark is accompanied by a precipitous fall in the number of detectable exonization events (Fig. 4a and Additional file 1: Figure S4).

Given previous findings that Alu-containing sequences can promote nuclear retention [30], we investigated whether upon UV irradiation the polysome-engagement of genes was affected by exonization [31]. Notably, after UV irradiation we identify a significant decrease in the expression levels of genes containing Alu-containing exonization events within the polysome fraction, as compared to whole cell RNA-seq expression (Fig. 4b, *p* < 8.50 × 10^−22^, Wilcoxon rank-sum test). Furthermore, the median strength of this depletion increases with inclusion percentage of the novel exons (Fig. 4c, *p* < 1.49 × 10^−06^, Wilcox rank-sum test).

We were next interested in investigating the type of genes in which exonization events are promoted after UV irradiation. Interestingly, there is a strong enrichment for genes involved in cell cycle regulation and RNA binding (Fig. 4d and Additional file 1: Figure S4). Altogether, these results suggest that DNA damage downregulates the ribosomal engagement of cell cycle genes partially through promoting exonization events containing Alu elements, which are known to induce nuclear retention [30]. Finally, we investigated if this was an evolutionarily conserved mechanism by evaluating rates of exonization upon UV irradiation in mouse embryonic fibroblasts. Remarkably, we identify a clear increase in exonization events in UV-treated versus untreated samples with the inclusion of rodent-specific B-repeats particularly affected (Additional file 1: Figure S4). Functional analysis of the genes containing novel exons once again revealed a strong enrichment for cell cycle genes (Additional file 1: Figure S4).

### Exonization is depleted in hematologic cancers

**Fig. 5:**
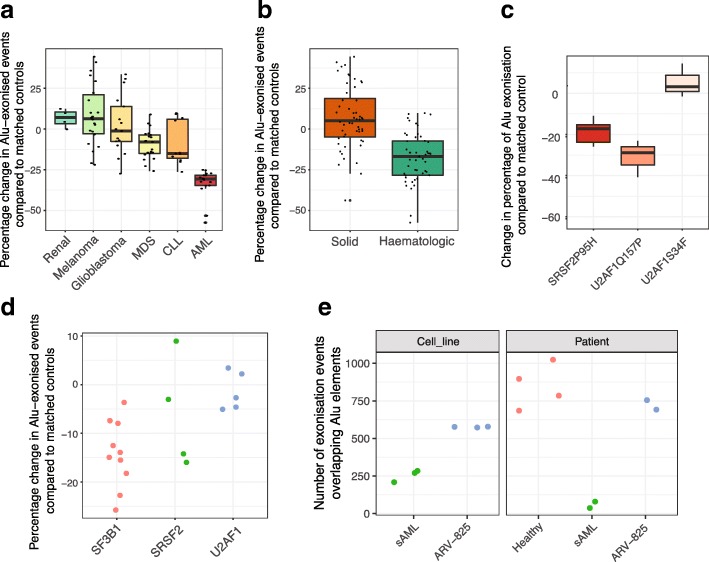
Hematologic cancers display decreased exonization. **a** Boxplots displaying percentage change in Alu-exonized events compared to matched patient controls. Dots represent data from individual samples. See Fig. 2c for the description of boxplots. SCLC, small cell lung cancer; MDS, myelodysplastic syndromes; CLL, chronic lymphocytic leukemia; AML, acute myeloid leukemia. **b** Boxplots showing data from **a** collated into two major cancer types. See Fig. 2c for the description of boxplots. **c** Boxplots displaying changes in Alu exonization relative to matched control samples in cell lines expressing RNA binding proteins with known cancer mutations. See Fig. 2c for the description of boxplots. **d** Dot plot displaying changes in Alu exonization of MDS samples grouped by genes containing mutations. Each dot represents data from an individual study. **e** Dot plot showing the number of exonized events in SET2 cell line samples and patient samples before and after administration of bromodomain inhibitor ARV-825. Independent healthy control samples from the same cell type (CD34+) are also included. ARV-825, BRD4 inhibitor

Aberrant splicing is a hallmark of cancer and contributes to numerous aspects of tumor biology [32]. Cancer-associated changes in splicing have been linked to altered expression of RBPs, some of which are oncogenic or act as tumor suppressors [33]. Despite extensive evidence for altered splicing in cancer, the extent to which these changes impact exonization has not been explored. We therefore evaluated the occurrence of exonization across matched tumor and control samples of patients within a variety of different cancers (Additional file 5: Table S4). Remarkably, this analysis revealed a significant and reproducible suppression of exonization within patient samples with chronic lymphocytic leukemia (CLL) and myelodysplastic syndromes (MDS) (Fig. 5a, CLL: *p* < 2.14 × 10^−04^; MDS: *p* < 1.15 × 10^−04^; AML: *p* < 4.02 × 10^−09^; Wilcoxon rank-sum test), which was independent of expression changes (Additional file 1: Figure S5). Moreover, this suppression of exonization is specific to hematologic malignancies (Fig. 5b, *p* < 2.93 × 10^−09^, Wilcoxon rank sum test). Consistent with our previous observations, the inclusion of novel exons is suppressed within genes associated with cell cycle and mRNA processing (Additional file 1: Figure S5 and Additional file 6: Table S5, *p* < 1 × 10^−5^, corrected FDR, compared to control samples).

Recent whole-genome-wide sequencing studies of patient samples with myelodysplastic syndromes have revealed frequent somatic mutations in a key group of spliceosome-associated proteins, including Serine/arginine-rich splicing factor 2 **(**SRSF2) and Splicing factor U2AF 35 kDa subunit.

(U2AF1) [10]. These mutations result in the mis-splicing of hundreds of transcripts [34–36]. Given previous work linking U2AF1 regulation and exonization events [9, 15], we explored if these types of mutations may help explain the suppression of exonization observed in the patient data. To investigate this possibility, we analyzed GRO-seq data from a HEK-293 cell line expressing wild-type (SRSF2 or U2AF1) or mutant splicing factors with (SRSF2(P95H), U2AF1(Q157P)) and without (U2AF1(S34F)) gain of splicing function mutations [37]. This analysis revealed that both the P95H mutation within SRSF2 and the Q157P mutation within U2AF1 inhibited the rate of novel exon inclusion (Fig. 5c, *p* < 1.46 × 10^−03^, Wilcox-rank sum test, compared to controls) while the S34F in U2AF1 had little effect. To investigate if these changes reflect the mutational load of the MDS patient samples, we grouped these data together based on genomic mutations within their RBPs. In agreement with cell line data, this analysis revealed a stratification of exonization based on the type of genomic RBP mutation (Fig. 5d and Additional file 1: Figure S5).

To investigate if this suppression of exonization in cAML could be relieved by decreasing the rate of transcriptional elongation, we evaluated the impact of the drug ARV–825, which is known to inhibit RNAPII elongation by promoting the degradation of Bromodomain-containing protein 4 (BRD4) [37]. The analysis of cAML patient samples replicated our previous results showing suppression of exonization (Fig. 5e). Importantly, this suppression is reversed upon application of BRD4 inhibitors with a 12-fold increase in the number of detected exonization events (Fig. 5e, *p* < 4.86 × 10^−91^, hypergeometric test). Altogether, this suggests in blood cancers exonization events within cell cycle genes is suppressed but can be strongly reversed by pharmacological intervention that increases the “window of opportunity”.

## Discussion

**Fig. 6:**
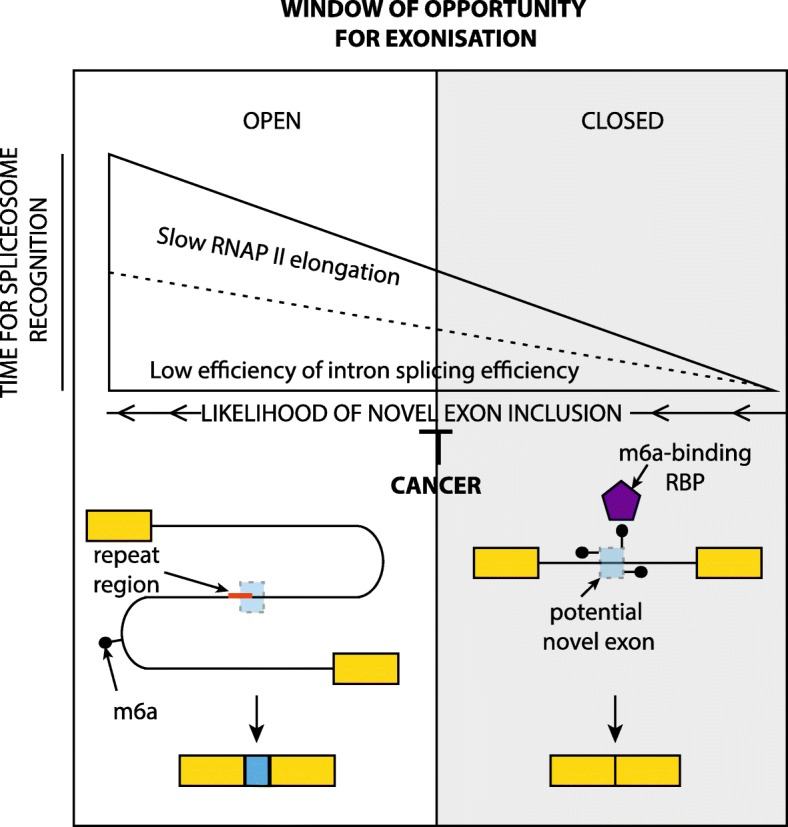
A model summarizing results from this paper. A model summarizing results from this paper contrasting regulatory mechanisms associated with opening (or facilitating) and closing (or inhibiting) the window of opportunity for exonization. RBP, RNA binding protein; RNAP II, RNA polymerase II; m6a, N^6^-methyladenosine

In this study, we show that exonization events arise within introns enriched with young transposable elements that are close to the transcription start site and overlap with nucleosome-binding sites. These processes promote exonization by extending the “window of opportunity” [25, 38] for the spliceosome to recognize novel exons. We highlight this can occur by decreasing the rate of RNAPII elongation and is associated with slow splicing efficiency dynamics. These novel exons are also marked by m6a RNA modifications. This multi-layered system permits exogenous forces to regulate exonization (Fig. 6). We demonstrate UV irradiation increases the rate of exonization within cell cycle genes, potentially by slowing RNAPII elongation [27, 28], and observe that exonization within these genes coincides with reduced polysome engagement. Furthermore, we describe in cancer how this “window of opportunity” mechanism is repressed and link this suppression to particular cancer mutations [34] within RNA binding splicing factors. Collectively, these results provide new insights into the control and dynamics of exonization in different biological and disease contexts, as well as highlighting an evolutionary process with the potential to expand regulatory complexity within cells.

Previous work has shown that RBPs and nucleosome occupancy underlie the regulatory control of exonization. For example, competition between the hnRNPC and the 3′-splicing factor U2AF2 [18], in tandem with nonsense-mediated decay [15], has been shown to restrict the inclusion of Alu-containing exons. On the other hand, high nucleosome occupancy is associated with the emergence of new exons [7, 16] and proposed to promote RNA polymerase II pausing [7, 16, 39]. Our observations expand these findings identifying a “window of opportunity” model [25, 38] for exonization controlled by a multi-layered regulatory program, including m6A-associated RBPs that suppress the emergence of new exons. The regulatory networks controlling exonization events are highly interconnected, as RNAPII facilitates the deposition of m6a onto actively transcribing nascent transcripts [40], which is known to tune splicing efficiency [21]. Given our observations that exonization is subject to multi-layered regulatory control (i.e., RNAP-II, RBPs and m6A—see Fig. 6), it is also interesting to consider how this mechanism may influence the life cycle of a transcript. Our results show exonization is associated with decreases in polysome association of genes containing exonization events. An explanation for this observation is that short sequences derived from Alu elements [30] and transcripts with other repeats [41, 42] have increased nuclear accumulation, which would restrict the ribosomal accessibility of transcripts with exonization events (Additional file 1: Figure S4).

It also noteworthy that a system likely evolved to suppress the aberrant impact of transposon inclusion on functional transcripts [43] may have been co-opted to create a novel regulatory mechanism. In support of this proposal, we identify UV irradiation is accompanied by an increase in exonization within cell cycle genes potentially restricting the expression of key checkpoint regulators, until the DNA damage process is complete. These changes may be the result of perturbations to the multi-layered regulatory network controlling exonization, for example, UV irradiation slows the elongation rate of RNA polymerase II [28], and DNA damage up-regulates the m6a-regulatory machinery [29] partially re-localizing hnRNPC to sites of DNA damage [44] which may impact its role in suppressing Alu recognition by the splicing machinery [15]. In contrast to the aforementioned system, in blood cancers, where increased mutational load is potentially advantageous for tumor progression [45], we observe that the inclusion of repeat-associated novel exons is suppressed potentially as a consequence of METTL3’s role as an oncogene and driver of AML [46]. Interestingly, in line with our “window of opportunity” model, we also demonstrate that in AML drugs reducing the speed of transcription elongation can reverse this suppression and promote exonization. This suggests a route of novel therapeutic intervention as increasing the rate of exonization in cancers may permit more expansive expression of tumor-specific antigens [47] thereby expanding the landscape of immunotherapeutic targets.

## Conclusions

In summary, we uncover extensive exonization events within repeat-associated intronic regions in various contexts, including m6A regulation, DNA damage induced regulation of cell cycle genes, and hematological cancer. We demonstrate exonization impacts ribosomal engagement of cell cycle transcripts in the context of DNA damage, highlighting a potential role of exonization in gene regulation. Altogether, we show that the multilayered regulation of exonization is a pliable process capable of expanding the transcriptomic complexity and regulatory capacity of cells.

## Methods

### Data processing

All fastq files were quality checked using FastqC [48]. Further quality checks were done using Trimmomatics [49] to remove adaptors, low-quality reads, and all reads less than 50 nucleotides in length.

### Datasets

All datasets used described in Additional file 5: Table S4 [2, 11, 21, 24, 28, 31, 50–65].

### Alternative splicing RNA-seq analysis

Whippet [12] was used to analyze RNA-Seq data employed for the identification of exonization events. Whippet quantifies all combinations of EEJs, including cassette, mutually exclusive, and microexon events. Whippet (v1.0) was run using default settings with “—biascorrect” option enacted to correct for 5´-sequence and GC content batch/bias errors (https://github.com/timbitz/Whippet.jl).

To create the splice graphs required for Whippet splicing quantification, genome annotation files were extracted from Ensembl (Hg38 – Release 93) [69]. For each dataset, this was supplemented with novel exon-exon junctions derived from whole-genome alignment by STAR [66] with 2-pass setting enabled and *outFilterMultimapNmax == 10*. Whippet index was run for each dataset with “—bam” setting enabled and “--suppress-low-tsl.” Whippet quantification using bias correction function enabled to correct 5′ sequence and GC content bias. Otherwise, the default settings were used, so that only reads mapping to exon-exon junctions were used to quantify splicing. Bedtools (intersect –v) [67] was used to remove all exons overlapping with annotation from UCSC genome browser [13]. UCSC Liftover [13] was used to convert all non-human exons in conservation analysis.

### Gene expression RNA-seq analysis

Kallisto [68] was used with default settings with index constructed using data extracted from Ensembl Hg38 Release 93 [69].

### Identification of exonization events

All events identified by Whippet were only considered as novel exonization events if they passed the following criteria: (1) Exon inclusion was supported by at least 5 corrected reads, as assigned by expectation maximization by Whippet. (2) Event must be CE (core exon) event. (3) Exon was not identified in any of the control/matched datasets. (4) Exon was not previously annotated in Ensembl Hg38 Gene transfer format (GTF) file or UCSC GTF file. (5) Exon had a percent-spliced in (PSI) value of at least 0.05 (i.e., 5%). (6) Exon-exon junction reads must occur between novel exon and known exon.

An exon was considered previously annotated if either exon-exon junction was annotated in Hg38 GTF file (from Ensembl or UCSC). The “number of exonization event” are all those events identified in this manner.

### Overlap with known repeat elements

Repeat elements identified by RepeatMasker were downloaded from UCSC table browser [13] in bed format. Bedtools intersect (−wao –f 0.2) was used to identify overlap of transposons with novel exons.

Frequency of Alu-transposable events is calculated as the proportion of exonization events overlapping transposons that are identified as Alu events. All Alu events identified by repeatmasker (containing annotation “Alu”) were grouped together.

### Visualization of events

Visualization of splicing events were done using –-bam setting for whippet quant and visualized with Sashimi plots in IGV browser (−DenableSashimi = “true”) [70].

### Functional analysis

Functional enrichment analysis was performed using the g:Profiler (https://biit.cs.ut.ee/gprofiler/gost) tool [71]. Genes identified as containing novel splicing events were compared to a background of genes expressed in sample (cRPKM or TPM > 1). Structured controlled vocabularies from Gene Ontology organization, as well as information from the curated KEGG and Reactome databases were included in the analysis. Only functional categorizes with more than five members and fewer than 2000 members were included in the analysis. Significance was assessed using the hypergeometric test. *p* values were corrected for multiple testing using the method of Benjamini-Hochberg. The Cytoscape application EnrichmentMap (baderlab.org/Software/EnrichmentMap) was used to visualize functional enrichment [72].

### General logistic regression

All continuous data was normalized to ensure fair comparison between features. The R module GLM with default setting except family = binomial(). Data was split into training and test data with 90:10% split. ROC curve calculated using test data using ROCR library.

### Exonic features

MaxEntScan [73] was used to estimate the strength of 3′ and 5′ splice sites. 5′ splice site strength was assessed using a sequence including 3 nt of the exon and 6 nt of the adjacent intron. 3′ splice site strength was assessed using a sequence including − 20 nt of the flanking intron and 3 nt of the exon. SVM-BPfinder [74] was used to estimate branchpoint and polyprimidine tract strength and other statistics. Score was estimated using the sequence of introns to the 3’end of exon between 20 and 500 nt.

Transcription start sites (TSS) were downloaded from Biomart. TAD boundaries for HepG2 were extracted from ENCODE [11] pre-processed data and converted to Hg38 by liftover. GC content was calculated using python script. Transposon information download from RepeatMasker as described above.

Nucleosome occupancy for HepG2 cells was calculated using data from Enroth et al. [75]. Colorspace read data was aligned using Bowtie [76] (-S -C -p 4 -m 3 --best –strata) using index file constructed from Ensembl Hg38. Nuctools (with default settings) was used to calculate occupancy profiles and calculate occupancy at individual regions [77].

In feature analysis, only exonization events within introns detected in this analysis were used.

### Splicing efficiency dynamics

Splicing efficiency dynamics was calculated using approach described previously [26]. Briefly, reads were mapped to Ensembl Hg38 assembly using STAR (2-pass enabled) and only uniquely mapped reads kept for downstream analysis. Splicing index values were first calculated which represent ratio of the split reads mapping to the 5′ and 3′ SJ of an intron divided to the sum of split plus non-split reads. The θ value (representing Splicing Efficiency, SE) was extracted from all pulse-chase time points, for introns with at least five reads coverage at both 5′ and 3′ SJ. K-means clustering used to identify give groups of distinct splicing efficiency (very fast, fast, medium, slow and very slow). The Splicing Efficiency Dynamics metric was calculated as SED = 1/ ((1.001 − *θ* 0 min) × (1.001 − *θ* 60 min)).

### Identification of m6a reads

After genome-wide mapping to Hg38 assembly using STAR (2-pass enabled) [66], CLAM (CLiP-seq Analysis of Multi-mapped reads) [22] was used to re-align multi-mapping reads and call peaks. CLAM realigner was used to assign multi-mapped reads in a probabilistic framework using expectation-maximization (----read-tagger-method start –retag). This was followed by CLAM peakcaller which call peaks by looking for bins enriched with IP reads over control, specifying a Negative-binomial model on observed read counts.

To calculate read coverage per nucleotide position the strand-specific “narrow_peak.combined.bed” produced by CLAM was converted to a bed file and then file was converted into wig files with bedtools genomecov using –scale to normalize for library size. bedtools coverage –d –s was used to identify depth per nucleotide. Overlap with transposable elements and novel exons was done using bedtools intersect (-f 0.2 –wao).

### Nascent RNA-seq analysis (including GRO-seq)

Wavefront and elongation speeds were extracted from supplementary data of relevant papers [23, 28].

### Investigation of influence of read depth on detection of novel exons

Reads were randomly sampled from a 100 M single-end HeLa RNA-seq dataset using the program “fastq-sample” from the “fastq-tools” (v0.8) pipeline using randomized seeds and no replacement. Identical pipeline (see “Identification of exonization events” section of methods) was run on every dataset and the percentage of Alu elements detected.

## Additional files

Additional file 1: **Figure S1.** Extension of Fig. 1 displaying the genomic features of introns associated with exonization events. **Figure S2.** Tissue-specific and shRNA analysis of novel exonic events. **Figure S3.** Extension of Fig. 3 displaying the analysis of the splicing efficiency dynamics. **Figure S4.** Extension of Fig. 4 showing evaluation of the UV exposure data in human and mouse samples. **Figure S5.** Extension of Fig. 5, and control analysis for detection of repeat elements at differing gene-expression levels and read depths. (PDF 2584 kb)

Additional file 2: **Table S1.** Genomic and splicing features used in logistic linear regression model. (XLSX 40 kb)

Additional file 3: **Table S2.** GO terms results for genes with exonization events. (XLSX 109 kb)

Additional file 4 Table S3. Coordinates, gene names and associated RNA binding knockdown identification for identified novel exon events.) (XLSX 764 kb)

Additional file 5: **Table S4.** Datasets used in paper including description and relevant figures information plus PubMed ID and SRA ID. (XLSX 54 kb)

Additional file 6: **Table S5.** GO terms results from GProfiler for exonization events identified depleted in cancer samples. (XLSX 254 kb)

Additional file 7: Review history. (DOCX 30 kb)

## Data Availability

All packages and publicly available computer code are described in the “Methods” section of this manuscript. Whippet is freely available at https://github.com/timbitz/Whippet.jl [12]. All plots were created using R Package ggplot and scripts are available upon requests. All data generated during this study are included in this article and its supplementary files or are available from external sources: all datasets analyzed in this study are available in SRA (list of identifiers and references provided in Additional file 5: Table S4 [2, 11, 21, 24, 28, 31, 50–65]). Additional in-house scripts with associated data-files are available in the Figshare repository [78].
