# Supplementary material for: Multilayered control of exon acquisition permits the emergence of novel forms of regulatory control
Source: Genome Biol. 2019 Jul 17;20:141. doi: 10.1186/s13059-019-1757-5 (PMC6637531; doi:10.1186/s13059-019-1757-5)
Supplement: Supplementary file 1 — Figure S1. Extension of Fig. 1 displaying the genomic features of introns associated with exonization events. Figure S2. Tissue-specific and shRNA analysis of novel exonic events. Figure S3. Extension of Fig. 3 displaying the analysis of the splicing efficiency dynamics. Figure S4. Extension of Fig. 4 showing evaluation of the UV exposure data in human and mouse samples. Figure S5. Extension of Fig. 5, and control analysis for detection of repeat elements at differing gene-expression levels and read depths. (PDF 2584 kb) [file 13059_2019_1757_MOESM1_ESM.pdf]

Supplementary Table Legends

Table S1: Genomic and splicing features used in logistic linear regression model

Table S2: GO terms results for genes with exonisation events

Table S3: Coordinates, gene names and associated RNA binding knockdown identification for identified novel exon events

Table S4: Datasets used in paper including description and relevant figures information plus pubmed ID and SRA ID.

Table S5: GO terms results from GProfiler for exonisation events identified depleted in cancer samples.

Supplementary Figures Legends

Fig. S1

a) Bar plot showing the fraction of Alu-mediated exonisation events identified upon knockdown of 6 RNA binding proteins (RBPs).
b) Receiver operating characteristic (ROC) curve of the accuracy of the logistic linear regression model for identifying introns containing exonisation events. Created using test data only.
c) Boxplots and barplots of selected features, as well as nucleosome occupancy, used as features in the logistic linear regression model. See Fig. 2d for description of boxplots. UTR – untranslated region. See method for details.
d) Enrichment map for GO, REACTOME, and KEGG functional categories of genes that contain exonisation events .(TOP) Fully annotated version of the enrichment map in Fig. 1b; (BOTTOM) enrichment map for genes overlapping L1 or Alu transposons. See Fig. 1b for description of enrichment maps

Fig. S2

a) A symmetrical heatmap of mutual pairwise similarities of data vectors as negative distances for percent spliced in (PSI) value of novel exons across multiple tissues (including testes, brain, heart, liver and kidney) across 4 primate species (human, chimp, gorilla and rhesus macaque). Vertical parallel heatmaps to right are colored by tissue and species types, respectively. These display that testes is the only tested tissue to cluster together. The rest of the tissues cluster primarily by species.
b) A barplot showing number of genes with conserved exonisation events across all four-primate species identified in a tissue.
c) Evaluation of shRNA-knockdown of over 200 RNA binding proteins in HepG2 cells from ENCODE database(11).
d) Barplots showing the number of exonisation events included upon knockdown of four RNA binding proteins. SRSF3 and YTHDC1 are known to bind m6a methylation marks. SRSF10 and SRSF9 have not been reported to directly bind m6a.
e) Boxplots displaying normalized intronic m6a peaks per nucleotide from nascent RNA in HepG2 cells. Non-exonised Alu elements occur in the same gene and therefore are controlled for expression level differences. Extension to Fig. 2C. See Fig. 2C for description of boxplots.

Fig. S3

a) Cumulative distribution plot showing the splicing efficiency dynamics for introns at different time-points in BrU-chase. Splicing efficiency is calculated by: split reads / (splilt reads + non-split reads). See Fig. 4b description of cumulative distribution plots.

b) Boxplot of splicing efficiency dynamics for introns with Alu-mediated exonisation events vs all expressed introns with no evidence of exonisation. Splicing efficiency dynamics is metric describing speed of intron excision at measured by assessing nascent RNA-seq using BrU-chase at 0, 15, 30 and 60 minutes. See Fig. 2d for description of boxplots. *** = $p < 1 \times 10^{-10}$

c) Extension of Figure 3B with an additional boxplot only including expressed introns from same gene as intron with exonisation events to control for differences in gene expression. See Fig. 2d for description of boxplots. *** = $p < 1 \times 10^{-10}$ P-value calculated using Wilcoxon-rank sum test. (n=4,011)

Fig. S4

a-b) Line plots showing the raw data for Fig. 4a

d) Sashimi plots of Alu-containing exonisation within G1/S1 checkpoint proteins E2F4 and LSM10. Displayed using IGV genome browser. Sashimi plots visualize splice junctions with width of lines representing number of mapped reads supporting exon-exon junction. In addition, Repeatmasker track from UCSC is displayed.

d) Bar plots showing the number of exonisation events in different cellular compartments, as well as the proportion of novel exons from each compartment, which overlap with transposons. Cyto = Cytosol; rep = repeat; 4SU = 4-thiouridine

e) Boxplots displaying percentage of all repeats elements (LEFT) and B_repeats (as a percentage of number of repeats: RIGHT) detected upon Ultraviolet (UV) irradiation compared to a control. See Figure 2c for description of boxplots

f) Functional analysis of genes with exonisation events up-regulated upon UV irradiation.

Fig. S5

a) Bar plot displaying functional analysis of genes with exonisation events in the patient control samples but absent in the MDS cancer samples. See Fig. 2c for description of boxplots. FDR = false discovery rate.

b) Dot plot displaying an extended version of Fig. 5d with genes divided into individual genomic mutations.

c) Heatmaps displaying gene expression in transcripts per millions (LEFT) and exonisation events in percent spliced in (RIGHT) for genes with novel exons identified in CLL lines or matched control datasets. Samples are clustered by hierarchical clustering in R.

d) Boxplots displaying the impact of read depth on Alu exonisation. All datasets were subsampled from the same parent RNA-seq sample with at least 3 repeats. The line plot displays the median. See Fig. 2c for description of boxplots

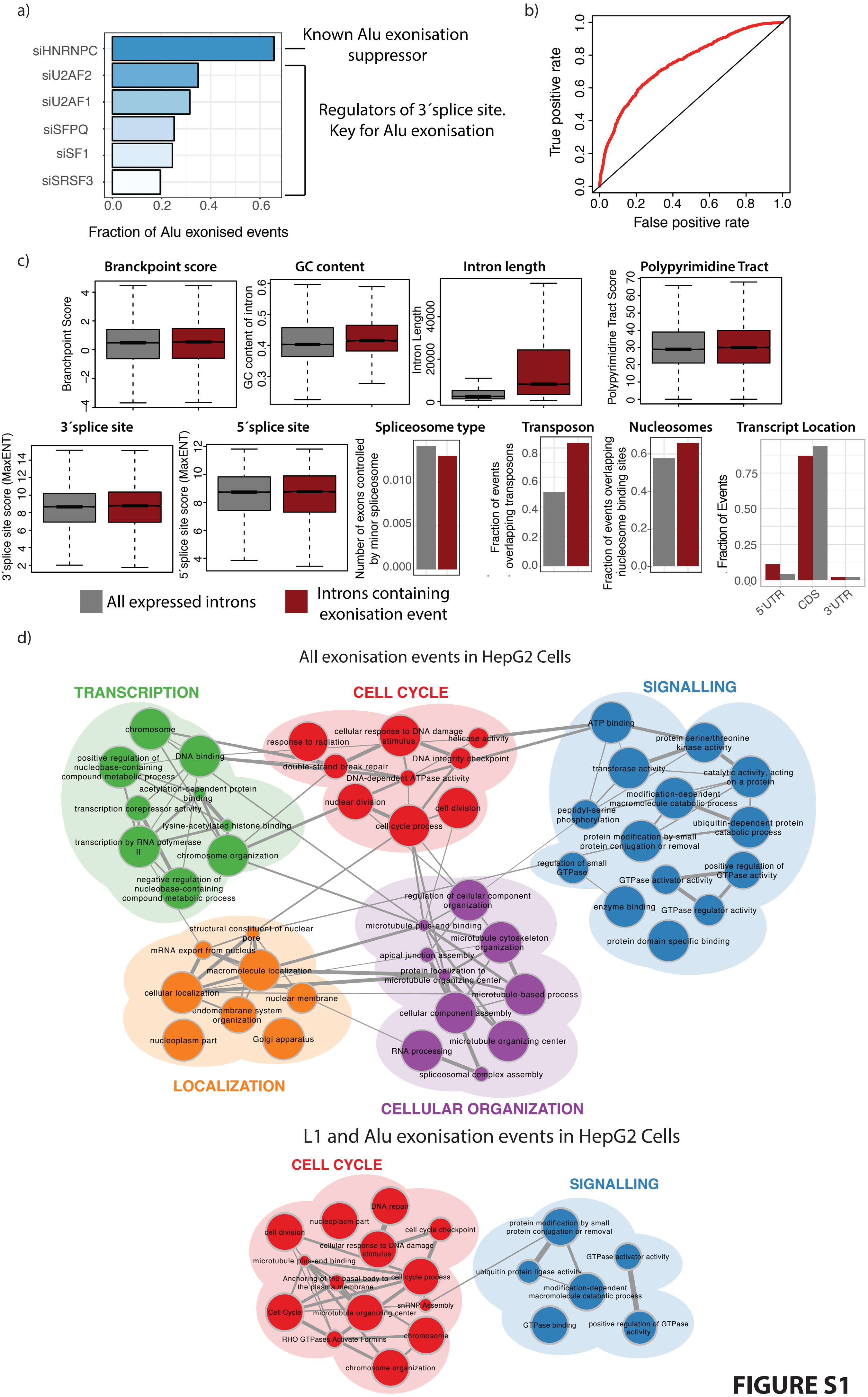
a)
siHNRNPC
siU2AF2
siU2AF1
siSFPQ
siSF1
siSRSF3
Fraction of Alu exonised events
Known Alu exonisation suppressor
Regulators of 3´splice site. Key for Alu exonisation
b)
True positive rate
False positive rate
c)
Branckpoint score
Branchpoint Score
GC content
GC content of intron
Intron length
Intron Length
Polypyrimidine Tract
Polypyrimidine Tract Score
3´splice site
3´splice site score (MaxENT)
5´splice site
5´splice site score (MaxENT)
Spliceosome type
Number of exons controlled by minor spliceosome
Transposon
Fraction of events overlapping transposons
Nucleosomes
Fraction of events overlapping nucleosome binding sites
Transcript Location
Fraction of Events
5'UTR
CDS
3'UTR
All expressed introns
Introns containing exonisation event
d)
All exonisation events in HepG2 Cells
TRANSCRIPTION
CELL CYCLE
SIGNALLING
LOCALIZATION
CELLULAR ORGANIZATION
L1 and Alu exonisation events in HepG2 Cells
CELL CYCLE
SIGNALLING

FIGURE S1

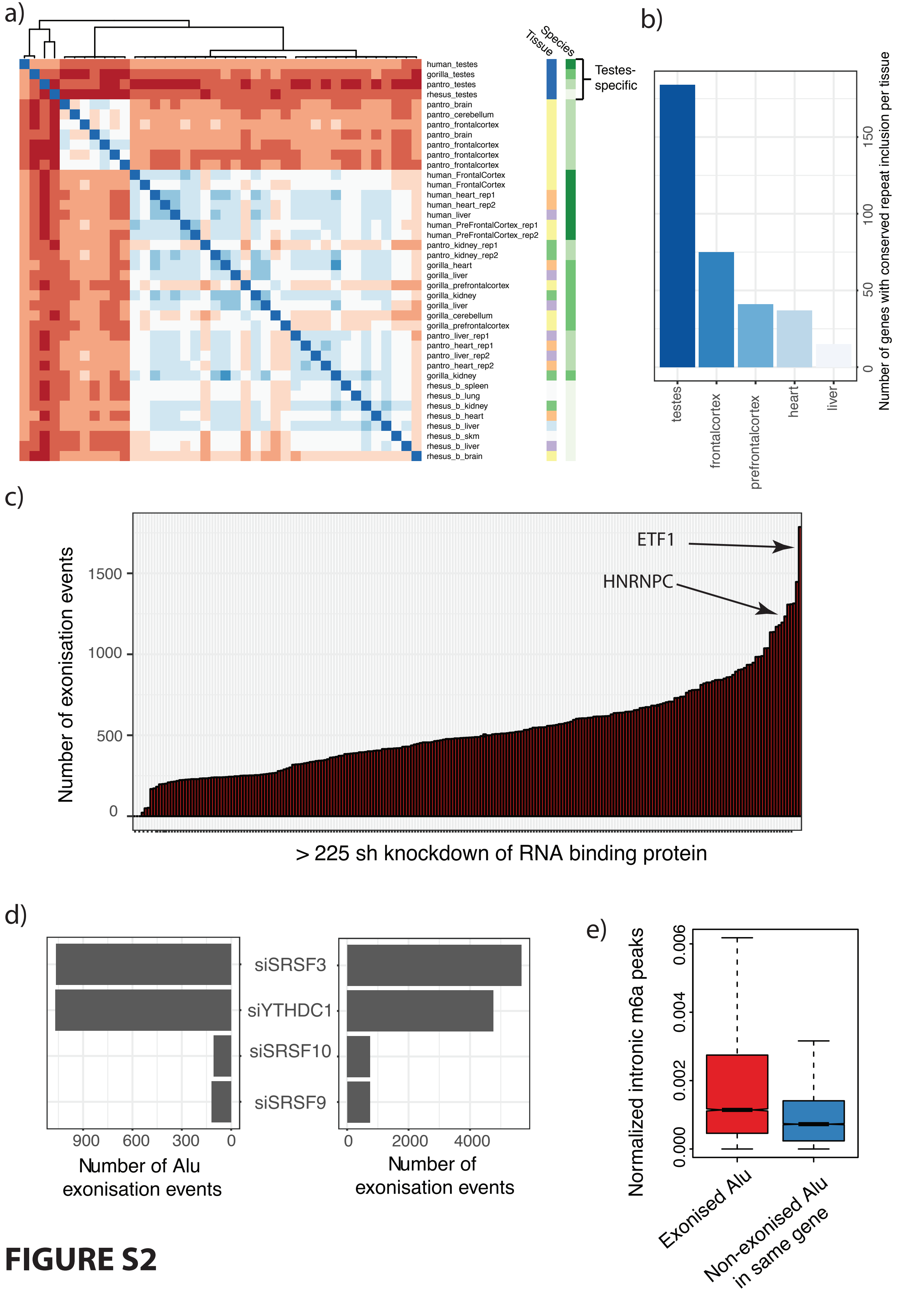
a)
Species
Tissue
Testes-specific
human_testes
gorilla_testes
pantro_testes
rhesus_testes
pantro_brain
pantro_cerebellum
pantro_frontalcortex
pantro_brain
pantro_frontalcortex
pantro_frontalcortex
pantro_frontalcortex
human_FrontalCortex
human_FrontalCortex
human_heart_rep1
human_heart_rep2
human_liver
human_PreFrontalCortex_rep1
human_PreFrontalCortex_rep2
pantro_kidney_rep1
pantro_kidney_rep2
gorilla_heart
gorilla_liver
gorilla_prefrontalcortex
gorilla_kidney
gorilla_liver
gorilla_cerebellum
gorilla_prefrontalcortex
pantro_liver_rep1
pantro_heart_rep1
pantro_liver_rep2
pantro_heart_rep2
gorilla_kidney
rhesus_b_spleen
rhesus_b_lung
rhesus_b_kidney
rhesus_b_heart
rhesus_b_liver
rhesus_b_skm
rhesus_b_liver
rhesus_b_brain
b)
Number of genes with conserved repeat inclusion per tissue
0
50
100
150
testes
frontalcortex
prefrontalcortex
heart
liver
c)
Number of exonisation events
0
500
1000
1500
ETF1
HNRNPC
> 225 sh knockdown of RNA binding protein
d)
siSRSF3
siYTHDC1
siSRSF10
siSRSF9
900
600
300
0
Number of Alu exonisation events
0
2000
4000
Number of exonisation events
e)
Normalized intronic m6a peaks
0.000
0.002
0.004
0.006
Exonised Alu
Non-exonised Alu in same gene

FIGURE S2

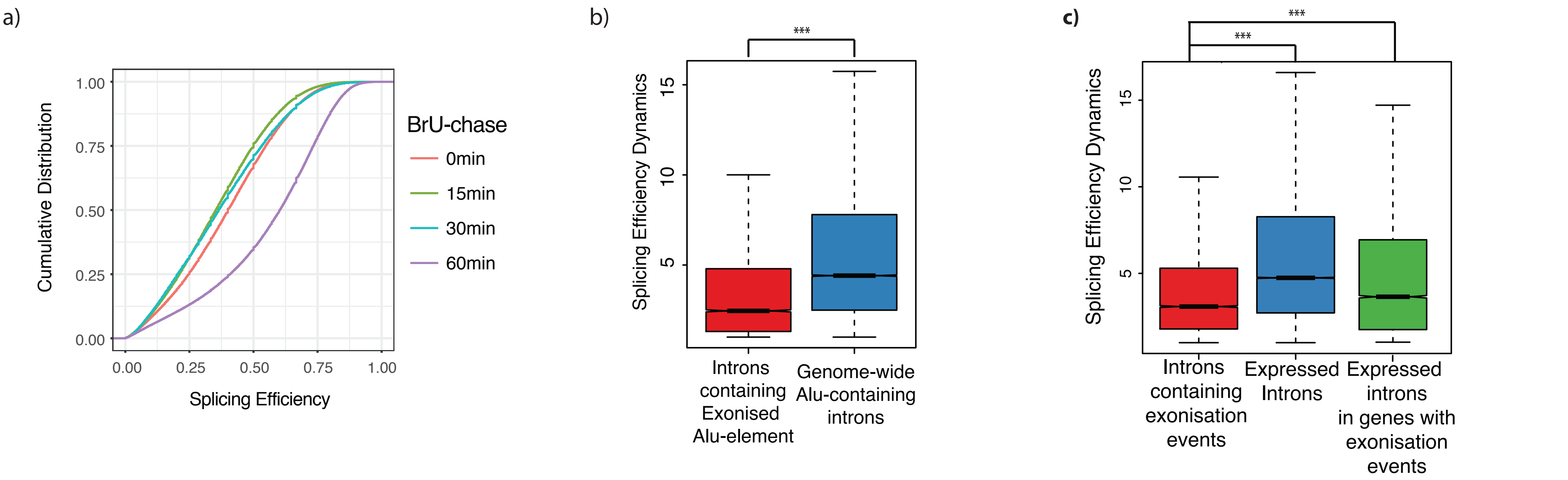
a)
Cumulative Distribution
1.00
0.75
0.50
0.25
0.00
0.00
0.25
0.50
0.75
1.00
Splicing Efficiency
BrU-chase
0min
15min
30min
60min
b)
***
Splicing Efficiency Dynamics
15
10
5
Introns containing Exonised Alu-element
Genome-wide Alu-containing introns
c)
***
***
Splicing Efficiency Dynamics
15
10
5
Introns containing exonisation events
Expressed Introns
Expressed introns in genes with exonisation events

FIGURE S3

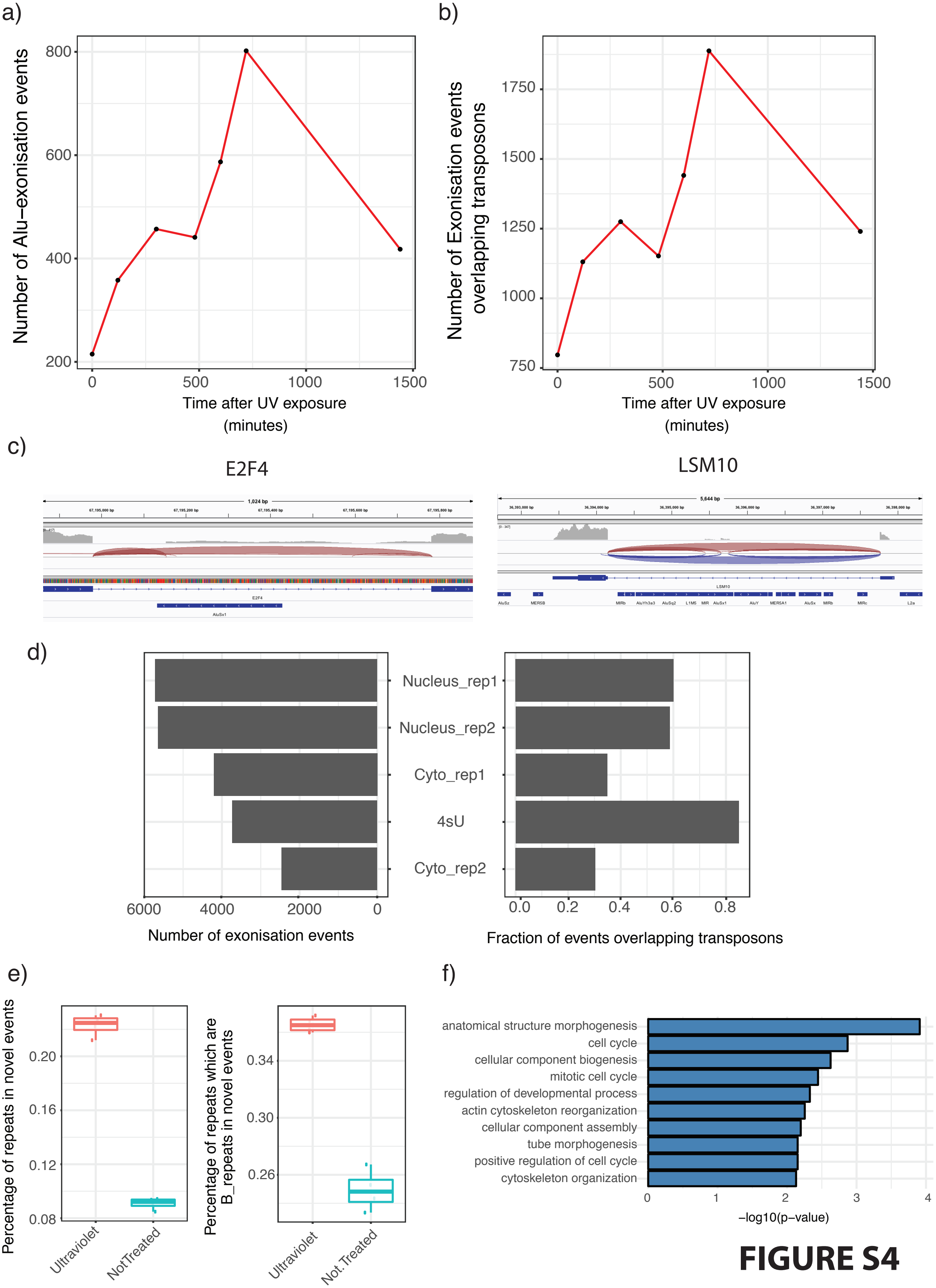
a)
Number of Alu−exonisation events
Time after UV exposure (minutes)
b)
Number of Exonisation events overlapping transposons
Time after UV exposure (minutes)
c)
E2F4
LSM10
d)
Nucleus_rep1
Nucleus_rep2
Cyto_rep1
4sU
Cyto_rep2
Number of exonisation events
Fraction of events overlapping transposons
e)
Percentage of repeats in novel events
Percentage of repeats which are B_repeats in novel events
Ultraviolet
NotTreated
Not.Treated
f)
anatomical structure morphogenesis
cell cycle
cellular component biogenesis
mitotic cell cycle
regulation of developmental process
actin cytoskeleton reorganization
cellular component assembly
tube morphogenesis
positive regulation of cell cycle
cytoskeleton organization
−log10(p−value)

FIGURE S4

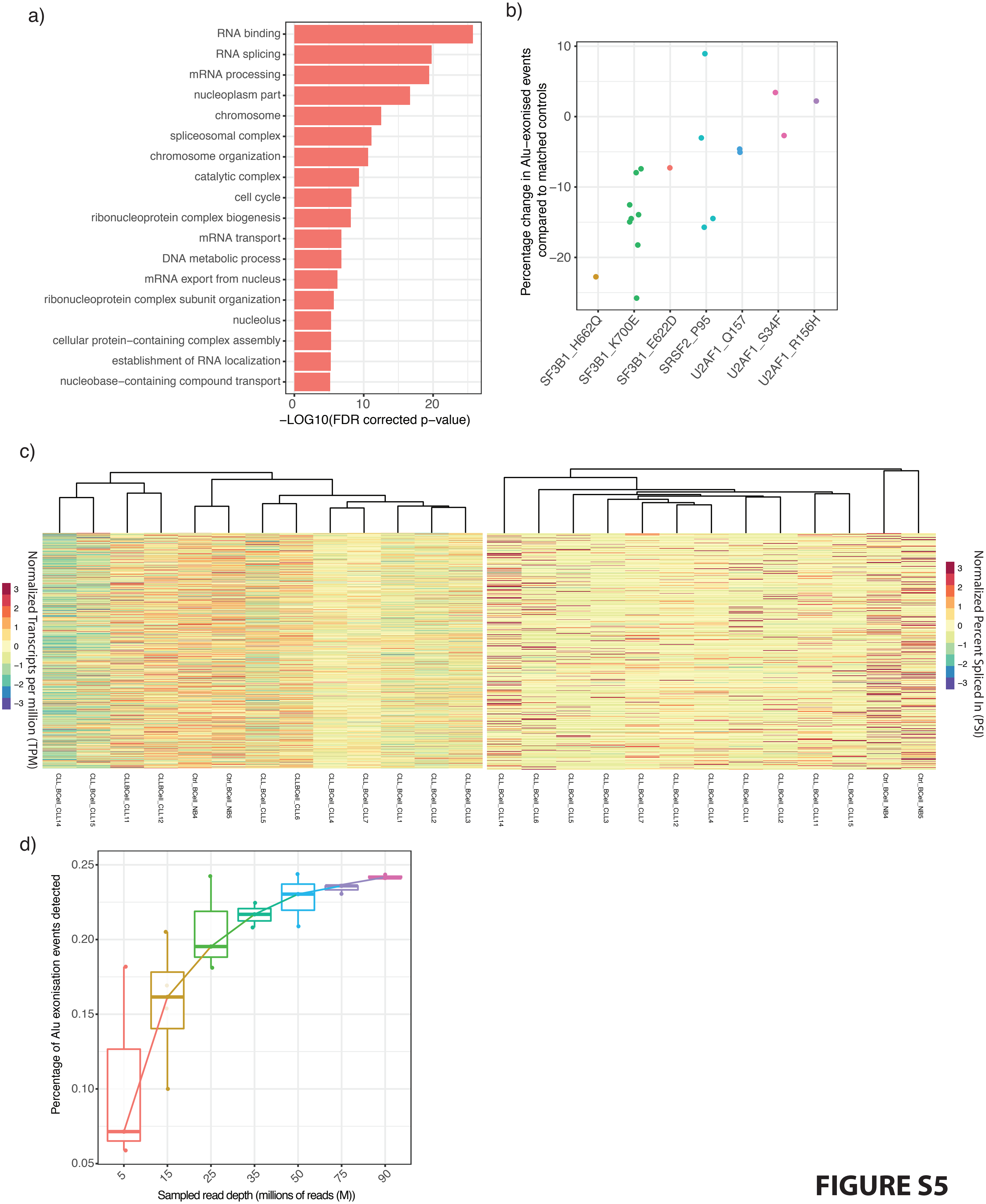

a)
RNA binding
RNA splicing
mRNA processing
nucleoplasm part
chromosome
spliceosomal complex
chromosome organization
catalytic complex
cell cycle
ribonucleoprotein complex biogenesis
mRNA transport
DNA metabolic process
mRNA export from nucleus
ribonucleoprotein complex subunit organization
nucleolus
cellular protein−containing complex assembly
establishment of RNA localization
nucleobase−containing compound transport
0
10
20
−LOG10(FDR corrected p−value)
b)
Percentage change in Alu−exonised events compared to matched controls
10
0
−10
−20
SF3B1_H662Q
SF3B1_K700E
SF3B1_E622D
SRSF2_P95
U2AF1_Q157
U2AF1_S34F
U2AF1_R156H
c)
Normalized Transcripts per million (TPM)
3
2
1
0
−1
−2
−3
CLL_BCell_CLL14
CLL_BCell_CLL15
CLLBCell_CLL11
CLLBCell_CLL12
Ctrl_BCell_NB4
Ctrl_BCell_NB5
CLL_BCell_CLL5
CLLBCell_CLL6
CLL_BCell_CLL4
CLL_BCell_CLL7
CLL_BCell_CLL1
CLL_BCell_CLL2
CLL_BCell_CLL3
CLL_BCell_CLL14
CLL_BCell_CLL6
CLL_BCell_CLL5
CLL_BCell_CLL3
CLL_BCell_CLL7
CLL_BCell_CLL12
CLL_BCell_CLL4
CLL_BCell_CLL1
CLL_BCell_CLL2
CLL_BCell_CLL11
CLL_BCell_CLL15
Ctrl_BCell_NB4
Ctrl_BCell_NB5
Normalized Percent Spliced In (PSI)
d)
Percentage of Alu exonisation events detected
0.25
0.20
0.15
0.10
0.05
5
15
25
35
50
75
90
Sampled read depth (millions of reads (M))

FIGURE S5
