# Supplementary material for: Multilayered control of exon acquisition permits the emergence of novel forms of regulatory control
Source: Genome Biol. 2019 Jul 17;20:141. doi: 10.1186/s13059-019-1757-5 (PMC6637531; doi:10.1186/s13059-019-1757-5)
Supplement: Supplementary file 7 — Review history. (DOCX 30 kb) [file 13059_2019_1757_MOESM7_ESM.docx]

Review History

**First round of review**

**Reviewer 1**

**Are you able to assess all statistics in the manuscript, including the appropriateness of statistical tests used?**

Yes, and I have addressed the statistics in my report.

**Comments to author:**

This study investigates the regulation and potential functional roles for "novel" exons in humans (referred to as exonisation events). The authors first use ENCODE RNA binding protein knockdowns to identify exonisation events (defined as non-annotated exons with significant inclusion in any of the knockdowns, but not in the controls). As expected, they find those exons to be enriched in transposable elements, especially Alus. Then, they used multiple diverse sources to investigate their regulation and function, coming up with a beautiful model for their potential impact in cell biology and in response to external queues. In brief, in addition to expected connections with RNA binding proteins (e.g. hnRNPC, U2AF1), they find that exonisation is repressed by m6A (presumably through m6A-binding proteins) and enhanced by slow RNApol2 elongation. Consistently, a slow RNApol2 mutant as well as UV treatment increases exonisation. Given that these exons are enriched in cell cycle genes, and their inclusion reduces their ribosome engagement, this is proposed to contribute/lead to reduction in proliferation. In line with these findings, they observe that exonisation is downregulated in certain cancers, potentially in line with mutations in U2AF1 (which should tip the scale towards hnRNPC dominance). Finally, they show this pattern can be reverted by a treatment enhancing RNApol2 elongation.

Given its novelty and potential biological impact, I believe this study is well suited for Genome Biology. Overall, I have only small concerns and a few suggestions for improvement:

1) I could not really understand why the authors decided to focus only on knockdown data to identify exonisation events. What is the physiological reason for this? Why not looking (also) in healthy tissues? In any case, I believe it would be very interesting to see how the identified events are regulated across tissues. One might expect that post-mitotic cells (particularly neurons) would have increased exonisation?

2) They required exonisation events not to be annotated in UCSC and ENSEMBL. Are they annotated in dedicated databases of alternative splicing (e.g. VastDB)?

3) For the comparison in Fig. 2c (and similar ones), is the control dataset matched for expression level? In other words, they mention they are "expressed genes", but could differences in expression (partly) explain the observed pattern?

4) I found Fig. 5e and the associated description in the main text a bit confusing, since the mix of patients and a cell line (+healthy for CD34+) makes it a bit confusing. Also, it may help to have the healthy samples first, then the sAML and then the rescue (from left to right in the X axis).

5) I missed a more explicit discussion on what can be known about the connection between UV treatment, recruitment of the m6A-associated machinery and exonisation. Does UV lead to increased m6A? What proteins of the machinery are recruited (if known)? How well does this all fit the author's regulatory model? It would be good to make all this explicit to avoid the reader having to dig into the primary sources to complete the story.

6) I suggest the authors make an additional figure with their model, to summarize the key concepts (genomic features, regulatory inputs, potential physiological roles, etc.).

7) One of the most exciting findings is, of course, the possibility that exonisation, through cell cycle modulation, impacts tumorogenesis. I suggest, if possible:

7a) Look into data for fast and slow replicating cells, if available.

7b) It could be very interesting to generate a sort of "exonisation score", combining the patterns of RNApol2 elongation-responding and/or cell cycle-associated events and see if such a score associates with replication (e.g. using the proliferation index) and, perhaps even more excitingly, if it is associated with patient survivability in haematologic tumors.

8) Perhaps a bit beyond the scope, but if the authors could find indications that this process is largely conserved in mouse (as a process, not the specific exons), it would be very exciting. Perhaps they can draw similar conclusions simply by identifying exonisation events in RNA-seq from UV treated cells.

**Reviewer 2**

**Are you able to assess all statistics in the manuscript, including the appropriateness of statistical tests used?**

Yes, and I have assessed the statistics in my report.

**Comments to author:**

The manuscript by Avgan et al. presents a detailed analysis of non-canonical or cryptic splicing events that result in inclusion of intronic splice site sequences as exons that are not known to form exons in healthy tissues as approximated by the comprehensive ENSEMBL and UCSC catalogues. The authors find that such exon-upon-perturbation events are particularly high in Alu retrotransposon exonisations; these are enriched towards cell cycle genes, and show increased inclusion after induction of DNA damage by UV. Finally, there is a disease aspect in that leukemias seem devoid of Alu-exons, linked to the high frequency of mutations in core spliceosomal factors in these tumours. An increase in Alu exons is seen upon ARV825 treatment, which suggests leukemic cancers could be hypersensitive to treatment inducing exonisation events and as a consequence, reduced cell cycle ability and increased proteome weaknesses.

A strength of the paper by Avgan et al. is the comparative assessment of classical exon definition as well as intronic and genomic features as qualifiers of such exons-upon-perturbation events across a significant number of perturbations from different sources; as well as their relationship to molecular mechanisms of exon definition such as RNA Pol II elongation speed and m6A modification rate. The computational and statistical analyses are sound, and the Methods are clearly detailed.

The big question that remains based on the presentation of the paper seems to be

- Are Alu exonisation events a regulatory feature of the DNA damage response that has been selected for fine-tuning the translation of cell cycle genes?

I believe the evidence by the authors is intriguing, but at this point remains speculative. The evidence is equally in line with an interpretation of Alu exonisation as a particular vulnerability of cell cycle genes. In other words, the authors have not addressed if this is a mistake a subset of genes is prone to, or a regulatory feature. Biochemical function (for instance decreased polysome association) does not equal cellular function & impact. Additional data would be needed to conclude that these events are functional. For instance, they need to be either (a) selected for during evolution or (b) necessary for the cellular response and there is a cellular defect if they are taken away.

On top, a few statements within the Discussion are over-statements that the authors do not need and should be rephrased. The manuscript also suffers at times from a lack of validation through independent or orthogonal secondary data, due to the restriction to data sets from the literature.

(Summary)

Therefore, I recommend substantial rephrasing of the manuscript, including the Abstract and Discussion. This should include a more balanced description of the advances made in the paper, focusing on the strengths of the analysis (exon-upon-perturbation at Alus, affecting a non-random set of genes, with relevance to biologically meaningful perturbations i.e. UV irradiation and cancer). The paper clearly outlines that de novo exonisation could be a regulatory feature in these scenarios but does not prove the hypothesis. Once the interpretation of the findings has been revised, I believe the paper will be suitable for publication and of interest to the Genome Biology readership.

- Jan Attig, Francis Crick Institute (London)

Major considerations

(A) Phrasing of findings in Abstract & Discussion

(i)

The authors wrote

Results

Here we observe that human genes are highly permissive to the inclusion of novel exonic regions permitting the emergence of novel regulatory features. We observe the potential for this to occur in over 20% of human genes.

This (subtly) suggests these 20% of human genes are qualitatively different from the other 80%. I heavily doubt they are. These 20% of human genes relate to the conditions that data was available for in this study (ENCODEs HepG2, UV, AML, etc). A substantial fraction of genes is quite tissue-specific, for instance neuronal, and from tissue-types where still few perturbation studies are done at large scale. Hence, the authors find exonisation-upon-perturbation events in 20% of genes that are easily accessible for such a survey. There will be a fraction of genes that genuinely do not ever show any exonisation-upon-perturbation, because they have very short introns such as cytokine genes. But the real number of exonisation-upon-perturbation events will be closer to 70-95% of human genes, so I recommend rephrasing this, either:

- state the number of genes with exonisation-upon-perturbation

and/or

- state the number of exonisation-upon-perturbation

and/or

- qualify the existing statement with 'under the perturbations we surveyed (short list)'

(ii)

"Conclusions

In summary, we show for the first time the extensive potential for exonisation to contribute to gene function and regulation."

This is far-fetched. With respect to Alu-exons, Gil Ast's work has characterized this as a path to expand gene function and regulation. Shen et al. showed Alu-exons can regulate the translation of mRNAs if placed in 5' UTRs in 2011 (PMID 21282640), L. Maquats and L. Chen/G. Carmichael have shown extensively that Alu-elements in 3' UTRs are regulating nuclear retention and mRNA translation; and the sentence stretches to every class of exonisation beyond Alu- and other retroelements.

Perhaps the authors meant to refer to internal (cassette) Alu-exons, for which there is limited evidence of biological significance. But still there is some, in particular Alu-exonisation events in proteins essential for homologous recombination in DNA repair after radiation, PMID 23585894; which potentially overlaps with the UV-induced events.

(iii)

choice of Title: "new forms of regulation"

I understand the authors try to convey emergence of novel regulatory features, but 'new forms' reads like absence of this form of control beforehand on the set of genes. For instance, absence of translational control at cell cycle genes without Alu exonisation. This obviously isn't the message of the paper, so I'd suggest a more precise phrasing. For instance: new entry points for regulation

(iii)

"into the control and function of exonisation in gene regulation" p15 line49

As outlined above, implication of a 'function' needs to be discussed appropriately.

(B) It is often unclear if exonisation of the same Alu elements in the same genes are detected in different conditions, or different Alu-elements in different genes.

Both are important observations, but need to be interpreted carefully. An increase in inclusion of the same Alu-elements suggests a regulatory feature at those elements (which the authors look at). The same is true for a change in exonisation of different Alu elements across the genome but this is more easily confounded by changes in expression of the genes that the Alu resides in, and by differences in the expression of regulatory factors and genomic features when comparing different cell lines/types. Hence, it is important for the reader to be able to deduce which comparisons are made.

- Figure 4A

"Alu inclusion" Is this the number of events (shown in Suppl Figure S4A) or the inclusion of those events? I assume the later, as the shape of the line graphs 4A and S4A look different. The inclusion of which exons is shown then in 4A? A combination of events from different time points totaling n = 3,278 ? Since that implies most exons were only detected in a single sample, why then present inclusion level instead of # of events?

Related 4C: we need to know the number of exons in each boxplot

- Figure 5:

Here I assume we are looking at the number of Alu-exonisation events. As this is inherently a function of sequencing depth, did the authors control for the sequencing depth of the tumour samples vs the matched normal samples? If so, where is this shown?

Fig. 5A. Given the spread and low sample number of data for Breast carcinoma and SCLC, I'd recommend removing these 2 indications. There seems no conclusion for these two indications that we could deduce.

Fig 5C. Similar, I'm confused as to what the number of Alu exons is that we are looking at here?

- Alu exonisation within cell cycle genes

Figure 1D, 4D and S5A show GO/KEGG pathway enrichments for cell cycle-related genes in three scenarios: knockdown of RNA-binding proteins, UV-irradiation, MDS patient data.

Could the authors present a venn diagram with the overlap of the genes affected by exonisation events? And comment on the # of Alu elements that exonise in more than one of the assays?

I am also wondering if cell cycle-associated genes are more prone to exonisation events, or just consistently among the highly expressed genes in different cell lines/types which creates a detection bias. Could the authors test features like expression level, intron number, intron length (etc.) through a linear regression similar to the initial exon-level analysis from Fig 1B; for groups of genes detected throughout the study? (cell cycle, RNA-binding / RNA processing vs a few control pathways of similar size such as proteasome, transcription).

(C) on m6A methylation

The authors discuss that knockdown of m6a RNA binding proteins increases exonisation, and among the occurring events, exonising Alus are enriched in m6A compared to other intronic Alus.

How does that relate to the remaining findings of the paper? Are m6A marks increased in cell cycle - DNA damage control genes ? Are slowly transcribed introns enriched in m6A mark? Are slowly transcribed introns more strongly bound by m6A binders in the ENCODE eCLIP data than expected by chance?

Some of the answers might be known from the literature, but the authors need to discuss the relevance of the m6A association they have revealed.

(D) More details are needed to interprete and maximize use of the data presented by the authors, particularly in Table S3

I believe the following details need to be provided:

- What is the number of genes across conditions that were expressed (say TPM>1 or a cut-off of the authors choice) that we can relate the exonisation events to? In other words, which percentage of genes covered had exonisation-upon-perturbation?

- related to: which introns are the "background group of expressed introns" (page 4 line 38)

- What is the (maximum observed) inclusion level of the exons in Table S3 in (one of) the conditions given in column E?

Minor requests

Description of figures

- Figure 4A / Supp Figure 4. Was the original GRO-seq experiment in single data points, without replicates? The figures appear to have no estimate of error/variation

- Figure 4c - I find the axis label a little confusing.

"Ribosome-associated genes" makes me think of genes encoding ribosomal proteins and associated factors. What the authors refer to is a polysome-association or translation efficiency score

- Figure label 2d and 5a refer to 2d but mean 2c

- Discussion, first paragraph

We demonstrate UV irradiation increases the rate of exonisation by slowing RNAPII

elongation(27, 28) suppressing cell cycle genes by reducing the polysome engagement of these UV-induced transcript variants. Furthermore, we describe in cancer how this mechanism is repressed and link this suppression to particular cancer mutations(34) within RNA binding splicing factors.

I assume the mutations in SF3B1 and U2AF1 in MDS/CML are known to reduce splicing efficiency, not RNA Pol II elongation speed. So the mechanism referred to is an extended window of opportunity of exon definition and competition, not slowing RNAPII elongation. This is unclear in the current phrasing.

- Figure label 5c is missing words

- page 13 line 60

"To investigate if these changes reflect the mutational load of the MDS patient samples, we grouped these data based on their genomic RBP mutations."

I understand within the context of the paragraph what the authors are trying to say but the sentence is rather cryptic.

**Authors Response**

**Point-by-point responses to the reviewers’ comments:**

*We thank both reviewers for their time and comments. We believe addressing these comments has greatly strengthened our paper.*

Reviewer #1: This study investigates the regulation and potential functional roles for "novel" exons in humans (referred to as exonisation events). The authors first use ENCODE RNA binding protein knockdowns to identify exonisation events (defined as non-annotated exons with significant inclusion in any of the knockdowns, but not in the controls). As expected, they find those exons to be enriched in transposable elements, especially Alus. Then, they used multiple diverse sources to investigate their regulation and function, coming up with a beautiful model for their potential impact in cell biology and in response to external queues. In brief, in addition to expected connections with RNA binding proteins (e.g. hnRNPC, U2AF1), they find that exonisation is repressed by m6A (presumably through m6A-binding proteins) and enhanced by slow RNApol2 elongation. Consistently, a slow RNApol2 mutant as well as UV treatment increases exonisation. Given that these exons are enriched in

cell cycle genes, and their inclusion reduces their ribosome engagement, this is proposed to contribute/lead to reduction in proliferation. In line with these findings, they observe that exonisation is downregulated in certain cancers, potentially in line with mutations in U2AF1 (which should tip the scale towards hnRNPC dominance). Finally, they show this pattern can be reverted by a treatment enhancing RNApol2 elongation.

Given its novelty and potential biological impact, I believe this study is well suited for Genome Biology. Overall, I have only small concerns and a few suggestions for improvement:

*We thank the reviewer for his/her interest in our work, and for the positive comments relating to our study.*

Reviewer #1 Major Comment 1:

I could not really understand why the authors decided to focus only on knockdown data to identify exonisation events. What is the physiological reason for this? Why not looking (also) in healthy tissues? In any case, I believe it would be very interesting to see how the identified events are regulated across tissues. One might expect that post-mitotic cells (particularly neurons) would have increased exonisation?

*Reviewer #1 Major Comment 1 Response:*

*The logic for focusing on knockdown data is twofold: (i) we are interested in understanding the impact of perturbations on novel exon inclusion (ii) we wish to have a clear control dataset.*

*However, to complement our original analysis, we fully agree that a tissue-wide evaluation of exonisation is an angle highly relevant to the interests of this study. In addressing this question, also in relation to Reviewer 2 Major Comment 1 in relation to evolutionary conservation of process, we undertook an analysis of exonisation events across tissues and primate evolution using data from Kaessmann and Burge labs (SRP007412 and SRP016501).*

*This analysis revealed that the testes are the tissue with the highest number of exonisation events, and this occurrence was conserved across primate evolution. We have added this analysis to Supplementary Figure 2A and B, as well as added the following text to the manuscript:*

*To evaluate the conservation of novel exon usage across species, we analyzed the extent of exonisation across multiple matched tissue types within four primate species spanning 30 millions years of primate evolution. To explore exonisation usage between samples, genes with events occurring in all four species were identified and sorted using affinity propagation clustering. In line with canonical alternative splicing {Barbosa-Morais et al., 2012, #18825; Merkin et al., 2012, #38635}, samples from the same species invariably clustered together (Supplementary Fig. 2). The notable exception to this trend was observed in samples from the testes, which showed tissue-specific clustering. This suggests within the testes there is a strong exonisation signature conserved across primate species in multiple genes (Supplementary Fig. 2).*

Reviewer #1 Major Comment 2:

They required exonisation events not to be annotated in UCSC and ENSEMBL. Are they annotated in dedicated databases of alternative splicing (e.g. VastDB)?

*Reviewer #1 Major Comment 2 Response:*

*We overlapped the identified novel exon events with those events annotated in VastDB (HsaEX events) and identified less than 1% of events overlapped with annotated VASTDB exons.*

Reviewer #1 Major Comment 3:

For the comparison in Fig. 2c (and similar ones), is the control dataset matched for expression level? In other words, they mention they are "expressed genes", but could differences in expression (partly) explain the observed pattern?

*Reviewer #1 Major Comment 3 Response:*

*The reviewer’s point is valid. We therefore have added an additional boxplot for both Figure 2C and Figure 3B only including introns from the same genes as exonisation events were detected. These analyses do not alter the conclusions drawn from the original analysis. These figures have been added to Supplementary Figures 2E and 3C.*

Reviewer #1 Major Comment 4:

I found Fig. 5e and the associated description in the main text a bit confusing, since the mix of patients and a cell line (+healthy for CD34+) makes it a bit confusing. Also, it may help to have the healthy samples first, then the sAML and then the rescue (from left to right in the X axis).

*Reviewer #1 Major Comment 4 Response:*

*Many thanks for the suggestions, we have also reworded the main text and have updated Figure 5 accordingly*

Reviewer #1 Major Comment 5:

I missed a more explicit discussion on what can be known about the connection between UV treatment, recruitment of the m6A-associated machinery and exonisation. Does UV lead to increased m6A? What proteins of the machinery are recruited (if known)? How well does this all fit the author's regulatory model? It would be good to make all this explicit to avoid the reader having to dig into the primary sources to complete the story.

*Reviewer #1 Major Comment 5 Response:*

*We have added two sections to the discussion to address these questions:*

*Our observations expand these findings identifying a “window of opportunity” model(25, 39) for exonisation controlled by a multi-layered regulatory program, including m6A-associated RBPs that suppress the emergence of new exons. The regulatory networks controlling exonisation events are highly interconnected, as RNAPII facilitates the deposition of m6a onto actively transcribing nascent transcripts (41), which is known to tune splicing efficiency (19).*

*and:*

*These changes may be the result of perturbations to the multi-layered regulatory network controlling exonisation, for example UV irradiation slows the elongation rate of RNA polymerase II (28), and DNA damage up-regulates the m6a-regulatory machinery (29) partially re-localizing hnRNPC to sites of DNA damage (45) which may impact its role in suppressing Alu recognition by the splicing machinery (14). In contrast to the aforementioned system, in blood cancers, where increased mutational load is potentially advantageous for tumour progression(46), we observe that the inclusion of repeat-associated novel exons is suppressed potentially as a consequence of METTL3’s role as an oncogene and driver of AML (47). Interestingly, in line with our “window of opportunity” model, we also demonstrate that in AML drugs reducing the speed of transcription elongation can reverse this suppression and promote exonisation.*

Reviewer #1 Major Comment 6:

I suggest the authors make an additional figure with their model, to summarize the key concepts (genomic features, regulatory inputs, potential physiological roles, etc.).

*Reviewer #1 Major Comment 6 Response:*

*We agree that this would be a nice addition to the paper. We have added a model diagram as Figure 6*

Reviewer #1 Major Comment 7:

One of the most exciting findings is, of course, the possibility that exonisation, through cell cycle modulation, impacts tumorogenesis. I suggest, if possible:

7a) Look into data for fast and slow replicating cells, if available.

7b) It could be very interesting to generate a sort of "exonisation score", combining the patterns of RNApol2 elongation-responding and/or cell cycle-associated events and see if such a score associates with replication (e.g. using the proliferation index) and, perhaps even more excitingly, if it is associated with patient survivability in haematologic tumors.

*Reviewer #1 Major Comment 7 Response:*

*The suggestion to investigate differences in fast and slow replicating cells is a very interesting one. However, we could only find datasets for mouse (Guo et al. 2014) that had collected RNA-seq from both slow and fast replicating cells. We undertook analysis on this dataset but found no major differences (data not shown).*

*We agree that an “exonisation score” would be a very interesting metric to create, however, we feel this is beyond the scope of this present work. We do however plan to extend this work in the future to include such a metric and to assess impact compared to patient survivability.*

Reviewer #1 Major Comment 8

Perhaps a bit beyond the scope, but if the authors could find indications that this process is largely conserved in mouse (as a process, not the specific exons), it would be very exciting. Perhaps they can draw similar conclusions simply by identifying exonisation events in RNA-seq from UV treated cells.

*Reviewer #1 Major Comment 8 Response:*

*We agree that this comment is a little beyond the scope of this paper but it is a rather interesting point and therefore we investigated it. Unfortunately, we were unable to find an identical dataset in mouse data but we did find data from mouse embryonic fibroblasts investigating the impact of UV damage upon p53 knockout. Our analysis shows a clear increase in exonisation events in the UV-treated versus not-treated samples (although the impact of knockout was negligible). Further investigation found the B-repeat events were particularly impacted by this regulation. Importantly, functional analysis revealed, once again, a strong enrichment for cell cycle-related genes.*

*We have added the following text to the manuscript:*

*Finally, we investigated if this was an evolutionarily conserved mechanism by evaluating exonisation inclusion upon UV irradiation in mouse embryonic fibroblasts. Remarkably, we identify a clear increase in exonisation events in UV-treated versus untreated samples with the inclusion of rodent-specific B-repeats particularly affected (Supplementary Fig. 4). Functional analysis of the genes containing novel exons once again revealed a strong enrichment for cell cycle genes (Supplementary Fig. 4).*

Reviewer #2:

The manuscript by Avgan et al. presents a detailed analysis of non-canonical or cryptic splicing events that result in inclusion of intronic splice site sequences as exons that are not known to form exons in healthy tissues as approximated by the comprehensive ENSEMBL and UCSC catalogues. The authors find that such exon-upon-perturbation events are particularly high in Alu retrotransposon exonisations; these are enriched towards cell cycle genes, and show increased inclusion after induction of DNA damage by UV. Finally, there is a disease aspect in that leukemias seem devoid of Alu-exons, linked to the high frequency of mutations in core spliceosomal factors in these tumours. An increase in Alu exons is seen upon ARV825 treatment, which suggests leukemic cancers could be hypersensitive to treatment inducing exonisation events and as a consequence, reduced cell cycle ability and increased proteome weaknesses.

A strength of the paper by Avgan et al. is the comparative assessment of classical exon definition as well as intronic and genomic features as qualifiers of such exons-upon-perturbation events across a significant number of perturbations from different sources; as well as their relationship to molecular mechanisms of exon definition such as RNA Pol II elongation speed and m6A modification rate. The computational and statistical analyses are sound, and the Methods are clearly detailed.

On top, a few statements within the Discussion are over-statements that the authors do not need and should be rephrased. The manuscript also suffers at times from a lack of validation through independent or orthogonal secondary data, due to the restriction to data sets from the literature.

(Summary)

Therefore, I recommend substantial rephrasing of the manuscript, including the Abstract and Discussion. This should include a more balanced description of the advances made in the paper, focusing on the strengths of the analysis (exon-upon-perturbation at Alus, affecting a non-random set of genes, with relevance to biologically meaningful perturbations i.e. UV irradiation and cancer). The paper clearly outlines that de novo exonisation could be a regulatory feature in these scenarios but does not prove the hypothesis. Once the interpretation of the findings has been revised, I believe the paper will be suitable for publication and of interest to the Genome Biology readership.

- Jan Attig, Francis Crick Institute (London)

*We thank Dr. Jan Attig for taking the time to review our work. His work has been seminal in this field and we therefore appreciate his support of our work and his constructive comments. Based on Dr. Attig’s comments, we have amended the text to provide a more balanced description of our findings, as well as fully addressed the rest of his comments.*

Reviewer #2 Major Comment 1:

I believe the evidence by the authors is intriguing, but at this point remains speculative. The evidence is equally in line with an interpretation of Alu exonisation as a particular vulnerability of cell cycle genes. In other words, the authors have not addressed if this is a mistake a subset of genes is prone to, or a regulatory feature. Biochemical function (for instance decreased polysome association) does not equal cellular function & impact. Additional data would be needed to conclude that these events are functional. For instance, they need to be either (a) selected for during evolution or (b) necessary for the cellular response and there is a cellular defect if they are taken away. The big question that remains based on the presentation of the paper seems to be - Are Alu exonisation events a regulatory feature of the DNA damage response that has been selected for fine-tuning the translation of cell cycle genes?

*Reviewer #2 Major Comment 1 Response:*

*Taking into consideration the concerns of the reviewer, we have revised the text taking particular precaution when using the word “function”. In addition, also based on Reviewer #1 Major comments 1 and 8, we assessed the conservation of these events during evolution. These analyses suggest that the patterns of exonisation are conserved within testes tissues across primate species (Reviewer Figure 1 and Revised Supplementary Figure 2A and B) and that upon UV irradiation in mouse MEFs, there is also an increase in exonisation within cell cycle genes (Reviewer Figure 5 and Revised Supplementary Figure 4E and F). We believe these findings imply repeat-derived exonisations, whether resulting from an escape of splicing repression or not, are conserved across evolutionary time increasing the likelihood that they may confer functionality.*

Reviewer #2 Major Comment 2:

(A) Phrasing of findings in Abstract & Discussion

(i)

The authors wrote

Results

Here we observe that human genes are highly permissive to the inclusion of novel exonic regions permitting the emergence of novel regulatory features. We observe the potential for this to occur in over 20% of human genes.

This (subtly) suggests these 20% of human genes are qualitatively different from the other 80%. I heavily doubt they are. These 20% of human genes relate to the conditions that data was available for in this study (ENCODEs HepG2, UV, AML, etc). A substantial fraction of genes is quite tissue-specific, for instance neuronal, and from tissue-types where still few perturbation studies are done at large scale. Hence, the authors find exonisation-upon-perturbation events in 20% of genes that are easily accessible for such a survey. There will be a fraction of genes that genuinely do not ever show any exonisation-upon-perturbation, because they have very short introns such as cytokine genes. But the real number of exonisation-upon-perturbation events will be closer to 70-95% of human genes, so I recommend rephrasing this, either:

- state the number of genes with exonisation-upon-perturbation

and/or

- state the number of exonisation-upon-perturbation

and/or

- qualify the existing statement with 'under the perturbations we surveyed (short list)'

*Reviewer #2 Major Comment 2 Response:*

*We agree with the reviewer’s point. In total, ~15,500 genes were detected (with TPM expression value > 1). We have changed the statement in results section to:*

*In total, we detected 13,103 novel exonic events within 4,774 genes or 30.6% of evaluated human protein-coding genes under the perturbations we surveyed.*

*and in the abstract:*

*Our analysis reveals the potential for novel exon acquisition to occur in over 30% of evaluated human genes.*

Reviewer #2 Major Comment 3:

"Conclusions

In summary, we show for the first time the extensive potential for exonisation to contribute to gene function and regulation."

This is far-fetched. With respect to Alu-exons, Gil Ast's work has characterized this as a path to expand gene function and regulation. Shen et al. showed Alu-exons can regulate the translation of mRNAs if placed in 5' UTRs in 2011 (PMID 21282640), L. Maquats and L. Chen/G. Carmichael have shown extensively that Alu-elements in 3' UTRs are regulating nuclear retention and mRNA translation; and the sentence stretches to every class of exonisation beyond Alu- and other retroelements.

Perhaps the authors meant to refer to internal (cassette) Alu-exons, for which there is limited evidence of biological significance. But still there is some, in particular Alu-exonisation events in proteins essential for homologous recombination in DNA repair after radiation, PMID 23585894; which potentially overlaps with the UV-induced events.

*Reviewer #2 Major Comment 3 Response:*

*We appreciate the reference of Anantha et al (2013), which we missed and have now included to our discussion. Our intention in the original text was to emphasize the extensive scale of exonisation events in the interrogated contexts. Unfortunately, this idea did not come across. We have therefore revised our text in the hope of reaching a solution that is in line with the references from the reviewer’s comment as below:*

*Our observations expand these findings identifying a “window of opportunity” model(25, 39) for exonisation controlled by a multi-layered regulatory program, including m6A-associated RBPs that suppress the emergence of new exons. The regulatory networks controlling exonisation events are highly interconnected, as RNAPII facilitates the deposition of m6a onto actively transcribing nascent transcripts (41), which is known to tune splicing efficiency (19).*

*Furthermore, we have changed the conclusion section as follows:*

*In summary, we uncover extensive exonisation events within repeat-associated intronic regions in various contexts, including m6A regulation, DNA damage induced regulation of cell cycle genes, and hematological cancer. We demonstrate exonisation impacts ribosomal engagement of cell cycle transcripts in the context of DNA damage, highlighting a potential role of exonisation in gene regulation. Altogether, we show that the multilayered regulation of exonisation is a pliable process capable of expanding the transcriptomic complexity and regulatory capacity of cells.*

Reviewer #2 Major Comment 4a:

choice of Title: "new forms of regulation"

I understand the authors try to convey emergence of novel regulatory features, but 'new forms' reads like absence of this form of control beforehand on the set of genes. For instance, absence of translational control at cell cycle genes without Alu exonisation. This obviously isn't the message of the paper, so I'd suggest a more precise phrasing. For instance: new entry points for regulation

*Reviewer #2 Major Comment 4a Response:*

*We have altered the title to include “novel forms of regulatory control”.*

Reviewer #2 Major Comment 4b:

"into the control and function of exonisation in gene regulation" p15 line49

As outlined above, implication of a 'function' needs to be discussed appropriately.

*Reviewer #2 Major Comment 4b Response:*

*we agree that word “function” has a multitude of biological connotations and therefore should be used carefully. We have therefore tried to limit our use of this word throughout the text. For example, we changed the sentence the reviewer highlighted to:*

*these results provide new insights into the control and dynamics of exonisation in different biological and disease contexts*

Reviewer #2 Major Comment 5:

It is often unclear if exonisation of the same Alu elements in the same genes are detected in different conditions, or different Alu-elements in different genes. Both are important observations, but need to be interpreted carefully. An increase in inclusion of the same Alu-elements suggests a regulatory feature at those elements (which the authors look at). The same is true for a change in exonisation of different Alu elements across the genome but this is more easily confounded by changes in expression of the genes that the Alu resides in, and by differences in the expression of regulatory factors and genomic features when comparing different cell lines/types. Hence, it is important for the reader to be able to deduce which comparisons are made.

*Reviewer #2 Major Comment 5 Response:*

*We agree with the general gist of the reviewer’s point but believe it is actually more extreme than they suggest. For example, major changes in gene expression between two perturbations would greatly increase the likelihood of novel exon detection just due to the increased expression of the gene (and independent of other factors we describe). To address this point and the general point of the reviewer, we assessed whether there were major changes in expression between datasets, which may explain our observations. We therefore matched genes in which our novel exonic events were identified with the gene expression (tpm) samples. In line with the changes in inclusion associated with many alternative-splicing events, the changes in percent spliced in (PSI) were independent of gene expression. Indeed, for gene expression events tested the control samples in this case did not even cluster independent of the cancer samples. We have added this analysis to the Supplementary Figure 5C, mentioned it in main text and have added the figure below for the reviewer’s convenience:*

Reviewer #2 Major Comment 6:

- Figure 4A

"Alu inclusion" Is this the number of events (shown in Suppl Figure S4A) or the inclusion of those events? I assume the later, as the shape of the line graphs 4A and S4A look different. The inclusion of which exons is shown then in 4A? A combination of events from different time points totaling n = 3,278 ? Since that implies most exons were only detected in a single sample, why then present inclusion level instead of # of events?

*Reviewer #2 Major Comment 6a Response:*

*In the hope of clearing off potential confusions Reviewer #2 has raised, we have made changes to the labelling of Figure 4A. The data describe the normalized number of Alu events as compared to the maximum, and the normalization process smoothens the shape of the line, in comparison to Supplementary Figure 4. The majority of events overlap and are seen in each sample. We have therefore changed the key in figure 4A.*

Reviewer #2 Major Comment 6b:

Related 4C: we need to know the number of exons in each boxplot

*Reviewer #2 Major Comment 6b Response:*

*We added the number of exons in each boxplot to the legend:*

*“Bin sample size from left to right: n = 427, 158, 355, 410 and 438.”*

Reviewer #2 Major Comment 7a:

- Figure 5:

Here I assume we are looking at the number of Alu-exonisation events. As this is inherently a function of sequencing depth, did the authors control for the sequencing depth of the tumour samples vs the matched normal samples? If so, where is this shown?

*Reviewer #2 Major Comment 7a Response:*

*The reviewer is correct and at low read depths (especially under ~25M) there is an inherent tendency for exonisation events to be detected with increases in read depth. However, above ~35-40M this association significantly weakens. All datasets were chosen to ensure an adequate read depth was available, especially in comparisons. For example, the CLL datasets average read depth 53.3 with a standard deviation of 11.4M. In fact the matched control datasets had a lower read depth of 40M and would therefore, if read depth had undue influence, have the opposing impact to what we found.*

*To investigate if indeed the influence of read depth plateaued after a certain read depth, we randomly sampled reads from a 100M single-end HeLa RNA-seq dataset using the program “fastq-sample” from the “fastq-tools” (v0.8) pipeline using randomized seeds and no replacement. Identical pipeline (see original methods) was run on every dataset and the percentage of Alu elements detected. This plot has been added to the supplementary figure 5D*

Reviewer #2 Major Comment 7b:

Fig. 5A. Given the spread and low sample number of data for Breast carcinoma and SCLC, I'd recommend removing these 2 indications. There seems no conclusion for these two indications that we could deduce. Fig 5C. Similar, I'm confused as to what the number of Alu exons is that we are looking at here?

*Reviewer #2 Major Comment 7b Response:*

*We have removed these samples in Fig. 5A and clarified Alu exons used.*

Reviewer #2 Major Comment 8a:

- Alu exonisation within cell cycle genes. Figure 1D, 4D and S5A show GO/KEGG pathway enrichments for cell cycle-related genes in three scenarios: knockdown of RNA-binding proteins, UV-irradiation, MDS patient data. Could the authors present a venn diagram with the overlap of the genes affected by exonisation events? And comment on the # of Alu elements that exonise in more than one of the assays?

*Reviewer #2 Major Comment 8a Response:*

*This data is available in Supplementary Table 1 and 5. Overlaps shows that ~44% (227/516) of cell cycle genes (n = 2,005) are similarly regulated between samples, which is greater than by chance (p < 1.19 x 10-8, Fisher exact test). However, controlling for variation in expression is difficult in this analysis. We are therefore reluctant to include a Venn diagram but are happy to include if required.*

Reviewer #2 Major Comment 8b:

I am also wondering if cell cycle-associated genes are more prone to exonisation events, or just consistently among the highly expressed genes in different cell lines/types which creates a detection bias. Could the authors test features like expression level, intron number, intron length (etc.) through a linear regression similar to the initial exon-level analysis from Fig 1B; for groups of genes detected throughout the study? (cell cycle, RNA-binding / RNA processing vs a few control pathways of similar size such as proteasome, transcription).

*Reviewer #2 Major Comment 8b Response:*

*Cell cycle genes are rarely among the most highly expressed genes. To demonstrate this, we did a comparison of cell cycle genes compared to other genes datasets, as defined by GO term annotation (see Figure below in uploaded document). The reviewer’s idea of a link between exonisation detection and high expression levels though is valid for RNA binding genes (hence why they were not investigated more), as hnRNP are among most highly expressed genes in many cells. However, cell cycle genes have 4th lowest median expression of the 11 function GO term categories tested.*

Reviewer #2 Major Comment 9:

on m6A methylation

The authors discuss that knockdown of m6a RNA binding proteins increases exonisation, and among the occurring events, exonising Alus are enriched in m6A compared to other intronic Alus.

How does that relate to the remaining findings of the paper? Are m6A marks increased in cell cycle - DNA damage control genes ? Are slowly transcribed introns enriched in m6A mark? Are slowly transcribed introns more strongly bound by m6A binders in the ENCODE eCLIP data than expected by chance? Some of the answers might be known from the literature, but the authors need to discuss the relevance of the m6A association they have revealed.

*Reviewer #2 Major Comment 9 Response:*

*We fully agree that our findings should be placed within the context of the numerous interesting papers in this field. We have therefore added the following section to the discussion section:*

*Our observations expand these findings identifying a “window of opportunity” model(25, 39) for exonisation controlled by a multi-layered regulatory program, including m6A-associated RBPs that suppress the emergence of new exons. The regulatory networks controlling exonisation events are highly interconnected, as RNAPII facilitates the deposition of m6a on actively transcribing nascent transcripts (41), which is known to tune splicing efficiency (19).*

*and:*

*These changes may be the result of perturbations to the multi-layered regulatory network controlling exonisation, for example UV irradiation slows the elongation rate of RNA polymerase II (28), and DNA damage up-regulates the m6a-regulatory machinery (29) partially re-localizing hnRNPC to sites of DNA damage (45) which may impact its role in suppressing Alu recognition by the splicing machinery (14). In contrast to the aforementioned system, in blood cancers, where increased mutational load is potentially advantageous for tumour progression(46), we observe that the inclusion of repeat-associated novel exons is suppressed potentially as a consequence of METTL3’s role as an oncogene and driver of AML (47). Interestingly, in line with our “window of opportunity” model, we also demonstrate that in AML drugs reducing the speed of transcription elongation can reverse this suppression and promote exonisation.*

*Finally, as requested in Reviewer #1 we have created a model figure to bring all our observations together (see Reviewer #1 Major Comment 6, Reviewer Figure 4. and revised Figure 6)*

Reviewer #2 Major Comment 10:

(D) More details are needed to interprete and maximize use of the data presented by the authors, particularly in Table S3

I believe the following details need to be provided:

- What is the number of genes across conditions that were expressed (say TPM>1 or a cut-off of the authors choice) that we can relate the exonisation events to? In other words, which percentage of genes covered had exonisation-upon-perturbation?

- related to: which introns are the "background group of expressed introns" (page 4 line 38)

- What is the (maximum observed) inclusion level of the exons in Table S3 in (one of) the conditions given in column E?

*Reviewer #2 Major Comment 10 Response:*

*We have provided the requested information and updated Table S3 accordingly.*

Reviewer #2 Minor requests 1

Description of figures

- Figure 4A / Supp Figure 4. Was the original GRO-seq experiment in single data points, without replicates? The figures appear to have no estimate of error/variation

*Reviewer #2 Minor requests 1 Response*

*The original GRO-seq data had replicates but to acquire the necessary read depth (see our answer to Reviewer #2 Major Comment 8), we were required to combine these datasets together.*

Reviewer #2 Minor requests 2

- Figure 4c - I find the axis label a little confusing.

"Ribosome-associated genes" makes me think of genes encoding ribosomal proteins and associated factors. What the authors refer to is a polysome-association or translation efficiency score

*Reviewer #2 Minor requests 2 Response*

*We have changed this phrase to “Ribosome-engaged genes”*

Reviewer #2 Minor requests 3

- Figure label 2d and 5a refer to 2d but mean 2c

*Reviewer #2 Minor requests 3 Response*

*We have corrected this error.*

Reviewer #2 Minor requests 4

- Discussion, first paragraph

We demonstrate UV irradiation increases the rate of exonisation by slowing RNAPII

elongation(27, 28) suppressing cell cycle genes by reducing the polysome engagement of these UV-induced transcript variants. Furthermore, we describe in cancer how this mechanism is repressed and link this suppression to particular cancer mutations (34) within RNA binding splicing factors.

I assume the mutations in SF3B1 and U2AF1 in MDS/CML are known to reduce splicing efficiency, not RNA Pol II elongation speed. So the mechanism referred to is an extended window of opportunity of exon definition and competition, not slowing RNAPII elongation. This is unclear in the current phrasing.

*Reviewer #2 Minor requests 4 Response*

*The reviewer is correct. We have corrected accordingly.*

Reviewer #2 Minor requests 5

- Figure label 5c is missing words

*Reviewer #2 Minor requests 5 Response*

*The reviewer is correct. We have corrected accordingly.*

Reviewer #2 Minor requests 6

- page 13 line 60

"To investigate if these changes reflect the mutational load of the MDS patient samples, we grouped these data based on their genomic RBP mutations."

I understand within the context of the paragraph what the authors are trying to say but the sentence is rather cryptic.

*The reviewer is correct. We have altered the wording of this sentence in hope of making this statement more comprehensible.*

*We thank the reviewers for their constructive feedback and helpful suggestions, which have helped us to produce a significantly improved manuscript.*

**Second round of review**

**Reviewer 1**

The authors have addressed all my concerns.

**Reviewer 2**

The authors have made a laudable effort to answer all questions/comments made by both reviewers. The major concerns have been addressed. The manuscript is a thoroughly prepared and ready for publication.
